# EEG Evidence Reveals Zolpidem-Related Alterations and Prognostic Value in Disorders of Consciousness

**DOI:** 10.3389/fnins.2022.863016

**Published:** 2022-04-27

**Authors:** Zexuan Hao, Xiaoyu Xia, Yang Bai, Yong Wang, Weibei Dou

**Affiliations:** ^1^Department of Electronic Engineering, Beijing National Research Center for Information Science and Technology (BNRist), Tsinghua University, Beijing, China; ^2^Department of Neurosurgery, The First Medical Center of PLA General Hospital, Beijing, China; ^3^Department of Neurosurgery, Hainan Hospital of PLA General Hospital, Sanya, China; ^4^Center for Cognition and Brain Disorders, The Affiliated Hospital of Hangzhou Normal University, Hangzhou, China; ^5^Key Laboratory of Intelligent Rehabilitation and Neuromodulation of Hebei Province, Department of Electrical Engineering, Yanshan University, Qinhuangdao, China

**Keywords:** EEG, disorders of consciousness, zolpidem, machine learning, prognosis, spectral analysis, functional connectivity, microstates

## Abstract

Effective treatment and accurate long-term prognostication of patients with disorders of consciousness (DOC) remain pivotal clinical issues and challenges in neuroscience. Previous studies have shown that zolpidem produces paradoxical recovery and induces similar change patterns in specific electrophysiological features in some DOC (∼6%). However, whether these specific features are neural markers of responders, and how neural features evolve over time remain unclear. Here, we capitalized on static and dynamic EEG analysis techniques to fully uncover zolpidem-induced alterations in eight patients with DOC and constructed machine-learning models to predict long-term outcomes at the single-subject level. We observed consistent patterns of change across all patients in several static features (e.g., decreased relative theta power and weakened alpha-band functional connectivity) after zolpidem administration, albeit none zolpidem responders. Based on the current evidence, previously published electrophysiological features are not neural markers for zolpidem responders. Moreover, we found that the temporal dynamics of the brain slowed down after zolpidem intake. Brain states before and after zolpidem administration could be completely characterized by the EEG features. Furthermore, long-term outcomes were accurately predicted using connectivity features. Our findings suggest that EEG neural signatures have huge potential to assess consciousness states and predict fine-grained outcomes. In summary, our results extend the understanding of the effects of zolpidem on the brain and open avenues for the application prospect of zolpidem and EEG in patients with DOC.

## Introduction

Effective treatment and accurate long-term outcome prediction remain challenging for disorders of consciousness (DOC) (Giacino et al., 2014; Edlow et al., 2021). Although major progress has been made in understanding DOC over the past decades, the extreme individual heterogeneity (e.g., etiology, location of impairment, and a clinical course) in patients with DOC and the heterogeneity of analysis methods and tools have strongly hampered our steps to find valid interventions and robust predictions of long-term recovery in patients with DOC (Pistoia et al., 2010).

Zolpidem, generally a sleeping pill, was first found to significantly improve consciousness in a patient who had been semi-comatose for 3 years (Clauss et al., 2000). This seemingly counterintuitive finding has quickly attracted the attention of both medical researchers and neuroscientists. To date, zolpidem has been found, essentially in case reports, to temporarily restore brain functions (e.g., cognition and motor) in patients with a variety of neurological disorders (Tucker and Sandhu, 2016; Bomalaski et al., 2017), including patients with DOC at the vegetative state/unresponsive wakefulness syndrome (VS/UWS) and minimally conscious state (MCS) with various etiologies (Clauss et al., 2000; Clauss and Nel, 2006; Cohen and Duong, 2008; Singh et al., 2008; Williams et al., 2013; Chatelle et al., 2014). In parallel with the above inspiring findings, results from larger sample (15–165 subjects) studies calm us down (Cohen and Duong, 2008; Thonnard et al., 2013; Du et al., 2014; Whyte et al., 2014; Zhang et al., 2021). Overall, only about 6% of patients with DOC responded positively and showed clinical improvement after zolpidem administration. Therefore, finding specific markers for zolpidem responders and uncovering other potential benefits of zolpidem (e.g., benefits for non-responders) are essential to enable the clinical use of zolpidem in patients with DOC. Given that the clinical features were largely indistinguishable between responders and non-responders (Zhang et al., 2021), and the presence of cognitive-motor dissociation in select patients with DOC (Edlow et al., 2021), neuroimaging techniques such as EEG can provide crucial information for identifying neural markers, exploring mechanisms, diagnosis, and prognosis (Giacino et al., 2014; Engemann et al., 2018; Arnts et al., 2020). However, only limited studies have used electrophysiological data to investigate zolpidem-induced alterations in patients with DOC. These are resting-state studies that are mainly based on spectral analysis and coherence/connectivity. Williams et al. (2013) showed that zolpidem administration sharply reduced EEG power/coherence at ∼6–10 Hz at some leads/lead pairs in all three patients with severe brain injury who were responders. Arnts et al. (2020) observed that an intact awareness patient with hypoxic-ischemic brain injury recovered his neurological function after zolpidem administration, with a decrease in beta band connectivity (corrected amplitude envelope correlation, cAEC). The EEG relative theta-alpha (4–12 Hz) power over the frontal and parietal cortices decreased, and the relative beta-gamma (15–50 Hz) power slightly increased after zolpidem administration. In addition, a recent magnetoencephalography (MEG) study has reported that a fully conscious, ex-coma patient with a traumatic brain injury that zolpidem strikingly improved his symptoms previously showed decreased power in the theta-alpha band (4–12 Hz), increased power in the high-beta–low-gamma band (20–43 Hz), and stronger connectivity (weighted phase lag index, PLI) between left frontal and temporal areas at 15–43 Hz after zolpidem, although, at the time of MEG measurement, the patient showed no clinical effects after zolpidem intake (Sripad et al., 2020). Briefly, prior studies on patients with brain injury have opened new doors for understanding the effects of zolpidem and showed decent agreement in theta-alpha power, but the results were inconsistent and difficult to compare in functional connectivity due mainly to the sample size and wide variation in the analysis methods, data modality, and etiology of patients. Furthermore, they all used static features and did not characterize the coordinated dynamics of the brain at a fast temporal scale (millisecond), albeit time-varying power spectra with 1-min resolution used in a study (Williams et al., 2013). The neural signal of the brain is non-stationary, and the brain dynamically transitions through distinct functional states, even at rest (Van de Ville et al., 2010; O’Neill et al., 2018; Zanesco et al., 2020; Park et al., 2021). Microstates have been described as the “atoms of thought” that underlie spontaneous conscious cognitive activity (Lehmann et al., 1998), resulting from the synchronous activity of brain networks (Zanesco et al., 2020). The major theories of consciousness suggest that the stream of consciousness is not continuous but consists of a series of quasi-stable brain-synchronized activities (Dehaene and Changeux, 2011; Michel and Koenig, 2018; Artoni et al., 2021). The microstate is an interpretation of this series of quasi-stable states (Michel and Koenig, 2018). A recent study has shown that microstate-related parameters can reflect the residual consciousness of patients with DOC (Gui et al., 2020). To date, numerous studies have been conducted on cognition and behavior in health and psychiatry based on the microstate method. However, currently, few studies explored the brain dynamics by microstate analysis in patients with DOC (Gui et al., 2020; Bai et al., 2021), and more research is needed to investigate and refine.

Despite the findings of the pioneering work (Williams et al., 2013; Arnts et al., 2020; Sripad et al., 2020), three questions need to be answered and refined. First, how do the EEG features of patients who show no clinical improvement alter after zolpidem administration? Do subgroups corresponding to different prognostic outcomes behave differently? Given that the current studies considered only responders (Pistoia et al., 2010; Bomalaski et al., 2017), one possible reason is the consideration of the clinical value and publication. However, the answer to this question is fundamental to identifying responder-specific neural markers. It is not reasonable to conclude that a feature is a responder-specific marker if it changes following the same pattern, regardless of whether the patient responds to zolpidem. Analogous to the anomaly detection problem, only knowing a considerable body of information about non-responders can better identify responders. Second, can the brain state of patients with DOC be distinguished before and after zolpidem administration using only EEG features? After severe brain injury, structural, functional, and metabolic disconnections in the brain can cause widespread dysfunction (Giacino et al., 2014; Salvalaggio et al., 2020; Edlow et al., 2021; Ros et al., 2022). If it is possible to distinguish the two brain states by EEG features, even in non-responders. This may provide new perspectives to distinguish the level of consciousness in patients with DOC and extend the potential benefits of zolpidem. Third, can baseline EEG features predict long-term prognostic outcomes (e.g., the coma recovery scale-revised score, the CRS-R score)? Accurate predictions are essential for guiding interventions and care planning. However, there is still a lack of studies using resting-state EEG for long-term fine-grained prediction (not the correlation analysis) of patients with DOC at the single-subject level (Giacino et al., 2014; Pauli et al., 2020; Song et al., 2020).

The major objective of the present study is to address the three questions above. Herein, we capitalized on multiple EEG analysis techniques, including spectral analysis, functional connectivity, and microstate analysis, to fully uncover how EEG features are altered by zolpidem in eight patients with non-acute DOC. We also investigated how neural features may be perturbed by DOC compared to healthy controls. On the one hand, we utilized spectral features and functional connectivity, some of which are like previous studies (Williams et al., 2013; Arnts et al., 2020; Sripad et al., 2020), to validate and extend the results of the prior reports. For spectral analysis, two types of features, relative power and the brain symmetry index (BSI), were calculated. Given the nature of inherently coordinated neural synchronization of brain function (Zanesco et al., 2020; Alnes et al., 2021; Carrasco-Gomez et al., 2021), a phase synchronization-based (phase-locking value, PLV) method (Lachaux et al., 1999) was used to compute the functional connectivity in five canonical frequency bands. On the other hand, we expanded from prior work using static features by exploring brain spatiotemporal dynamics using microstate analysis. Abundant microstate features were investigated in this study. Furthermore, we not only performed the analysis at the group and subgroup levels but also observed changes in each patient. To classify the status of patients with DOC before and after zolpidem administration and to predict the long-term outcome using EEG features, support vector machine (SVM) and support vector regression (SVR) models were constructed, respectively.

To our knowledge, such a comprehensive EEG analysis (integrated of static, dynamic, multilevel, multiband, classification, and prediction analysis) has not before been previously reported in patients with DOC on zolpidem. This would extend our understanding of the action mechanism of zolpidem on the brain and complex functional alterations in DOC conditions. It would also offer new insights into an accurate long-term prognosis, which is of growing clinical need.

## Materials and Methods

### Subjects and Clinical Evaluation

Eight patients with DOC (*M* = 47.9 years; *SD* = 9.9 years; range = 31–64 years) were enrolled in this study. The mean time from the brain injury was 4.5 months (range = 3–12 months). Detailed demographic and clinical information about patients with DOC are presented in Table 1. We also used a resting-state EEG dataset (eyes closed) of eight matched healthy controls (*M* = 47.8; *SD* = 8.9 years; range = 31–60 years), see Supplementary Table 1. The CRS-R score and diagnosis were evaluated for each patient with DOC before zolpidem administration and at the end time of the 6-month follow-up (T_*end*_). The evaluation was repeated at least three times within a week to avoid potential errors, owing to fluctuations in responsiveness. Eight patients were divided into two subgroups with improvement (Subgroup_I) and with non-improvement (Subgroup_N) based on the 6-month follow-up diagnosis compared to the diagnosis before zolpidem administration, and each subgroup contained four subjects (Table 1). Written informed consent was received from the legal representatives of patients and healthy controls. This study was approved by the Ethics Committee of the PLA General Hospital (Protocol No: 2017–33).

**TABLE 1:** Demographics and clinical information of patients with disorders of consciousness (DOC).

Patient ID	Gender	Age (years)	Etiology	Time to EEG (months)	CRS-R all/sub score (baseline)		CRS-R all/sub score (T_*end*_)		Diagnosis (baseline)	Diagnosis (Tend)	Subgroup	Patient alias
1	Male	49	Trauma	12	12	315102	22	456223	MCS+	EMCS	I	I1_T_MSC+
2	Male	47	Anoxia	3	7	112102	12	315102	VS/UWS	MCS+	I	I2_A_VS
3	Male	31	Stroke	3	7	112102	8	113102	VS/UWS	MCS−	I	I3_S_VS
4	Female	64	Trauma	3	7	103102	12	315102	MCS−	MCS+	I	I4_T_MSC−
5	Male	57	Stroke	4	6	102102	6	102102	VS/UWS	VS/UWS	N	N1_S_VS
6	Male	39	Stroke	4	6	102102	6	102102	VS/UWS	VS/UWS	N	N2_S_VS
7	Male	54	Stroke	4	10	133102	10	133102	MCS−	MCS−	N	N3_S_MSC−
8	Female	42	Trauma	3	6	112101	7	112102	VS/UWS	VS/UWS	N	N4_T_VS

*VS/UWS, vegetative state/unresponsive wakefulness syndrome; MCS−, minimally conscious state without language; MCS+, minimally conscious state with language; EMCS, emergence from MCS.*

*I, Subgroup_I; N, Subgroup_N.*

*The patient’s alias consists of the patient’s serial number in Subgroup_I or Subgroup_N, the etiology, and the baseline diagnosis.*

### EEG Acquisition and Preprocessing

**FIGURE 1:**
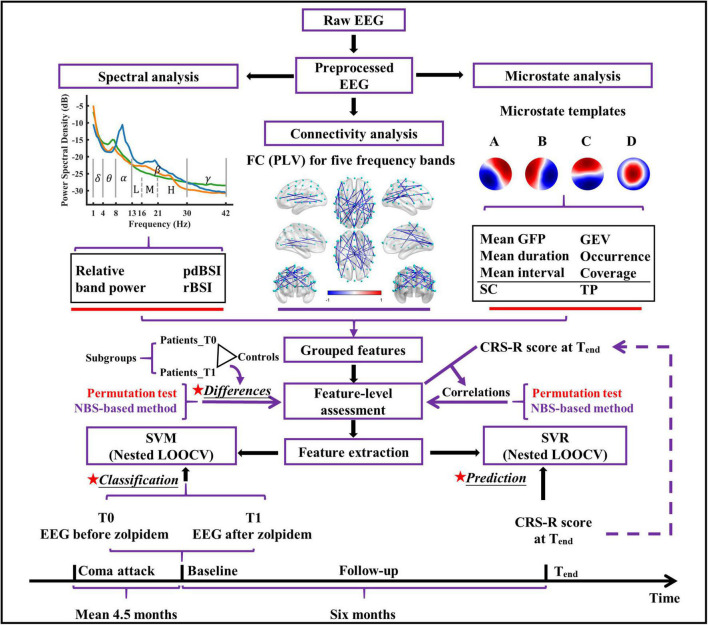
The conceptual framework of the present study. pdBSI, pairwise-derived brain symmetry index; rBSI, revised brain symmetry index; FC, functional connectivity; PLV, phase locking value; GFP, global field power; GEV, global explained variance; SC, spatial correlation metric; TP, transition probability; NBS, network-based statistic; SVM, support vector machine; SVR, support vector regression; CRS-R, coma recovery scale-revised; LOOCV, leave-one-out cross-validation.

The conceptual framework of this study is demonstrated in Figure 1. The EEG data of the patients were recorded from 59 scalp channel acquisition equipment (BrainAmp 64 MR plus, Brain Products, Munich, Germany), with the electrodes (Ag/AgCl) arranged in the standard international 10–10 system (see Supplementary Figure 1) and at a sampling rate of 2,500 Hz. The ground and reference electrodes were located at AFz and FCz, respectively. A low-pass filter (cutoff frequency = 250 Hz) was used in the EEG recorder. The electrode impedance was maintained below 5 kΩ during the entire acquisition process. At approximately 2 p.m., resting-state EEG data were collected for approximately 10 min before zolpidem administration for each patient. EEG data were also collected for approximately 70 min immediately after zolpidem administration (10 mg; SANOFI WINTHROP INDUSTRIE). To avoid interference with the experimental results, arousal procedures were not performed if the patient showed signs of drowsiness after zolpidem intake. Information of EEG acquisition for healthy controls is described in Supplementary Material.

Offline analysis was performed in MATLAB R2020b (Mathworks, Natick, MA, United States) using the EEGLAB toolbox^1^ (version 2019.0, Swartz Center for Computational Neuroscience, San Diego, CA, United States) and its extensions in combination with some custom MATLAB scripts. The EEG data of the patients and healthy controls followed the same preprocessing procedure. All preprocessing steps maintained double-precision computations. All filters used in this study were hamming-windowed finite-impulse response (FIR) filters designed using the *pop_firws* function. EEG data before zolpidem administration (T0) and 20–40 min after administration (T1) were selected and preprocessed. Previous studies have shown (significant) improvement, commencing 20–30 min after zolpidem in zolpidem responders, and thus the signal 20–40 min after zolpidem intake was selected in the study (Shames and Ring, 2008; Arnts et al., 2020).

First, EEG data were low pass filtered (order: 660, transition width: 5.0 Hz, a −6 dB cutoff frequency: 45 Hz), and down-sampled to 250 Hz, and then high-pass filtered (order: 826, transition width: 1 Hz, a −6 dB cutoff frequency: 0.5 Hz). Next, bad channels and segments containing excessive artifacts, if any, were removed by a semi-automatic code. Then, the signals of the removed bad channels were restored using spherical interpolation, and the EEG data were re-referenced to the common average. Finally, the EEG data were decomposed using independent component analysis (ICA), and the artifactual components (e.g., eye blinks, movement, channel noise, muscle activity, and heart) were identified and removed by visual examination and the ICLabel plugin. The EEG preprocessing metrics (number of removed channels, rejected components, and the length of EEG retained) for patients and healthy participants are summarized in Supplementary Table 2.

### Spectral Analysis

For each EEG recording, the power spectral density (PSD) was calculated using Welch’s periodogram with the Hamming window and 50% overlap. The window length was 2 s (500 samples, frequency resolution: 0.5 Hz), and windows with discontinuity events caused by artifact removal were not considered in the computation. For each channel, relative power in the delta (1.0–3.5 Hz), theta (4.0–7.5 Hz), alpha (8.0–12.5 Hz), low beta (13.0–15.5 Hz), middle beta (16.0–20.5 Hz), high beta (21.0–29.5 Hz), beta (13.0–29.5 Hz), and gamma (30.0–42.0 Hz) bands was measured. The relative power was calculated as the ratio of the summed absolute power in a particular band to summed absolute power over the 1.0–42.0 Hz range. The global relative power for each band was computed by averaging the channel-level relative power across all the channels. In addition, the global relative PSD (mean PSD across all channels divided by its summed values across the 1.0–42.0 Hz range) was calculated for each EEG recording. Pairwise-derived BSI (pdBSI) and revised BSI (rBSI) were calculated using PSD for the same frequency bands as the relative power (Mane et al., 2019; Saes et al., 2019). The basic distinction between pdBSI and rBSI is that pdBSI calculates the symmetry for each homologous channel pair before averaging across all channel pairs, whereas rBSI calculates the average PSD of the left and right hemispheres before calculating the symmetry.

### Functional Connectivity

Previous studies have shown that functional connectivity can be used as a reliable biomarker of neurological function (Wu et al., 2015) and to predict the severity and prognosis of comatose patients (Alnes et al., 2021; Carrasco-Gomez et al., 2021). Herein, a phase-based approach (PLV) of functional connectivity was performed. The preprocessed data were first applied to the surface Laplacian transform using the CSD toolbox (Kayser and Tenke, 2006) to minimize the volume-conducted effects. Then, EEG data were filtered into delta (1.0–4.0 Hz), theta (4.0–8.0 Hz), alpha (8.0–13.0 Hz), beta (13.0–30.0 Hz), and gamma (30.0–42.0 Hz) frequency bands, respectively, to compute the functional connectivity. The instantaneous phase was extracted by applying the Hilbert transform to each band-filtered EEG data, and 10% of the phase signals on each side were discarded to eliminate the edge effects. To better detect phase synchronization in non-stationary signals, we segmented the signal into 2 s non-overlapping epochs and calculated connectivity over time for each epoch (Hurtado et al., 2004), and then averaged the connectivity across epochs. For example, the PLV of channels *i* and *j* was computed by using the following formula:


$$PLV_{i,j}=\frac{1}{M}\sum_{m=1}^{M}\frac{1}{N}\left|\sum_{n=1}^{N}e^{-i\left(\varphi_{jn}-\varphi_{in}\right)}\right|,$$

where *M* is the number of epochs; *N* is the number of data points in one epoch, and φ_*i*_, φ_*j*_ are phase angles from channels *i* and *j.* A 59 × 59 matrix with the results of PLV was obtained in the end for each band.

### Microstate Analysis

Microstate analysis was performed to reveal the spatiotemporal dynamics of the resting brain. It was conducted in MATLAB with some custom-written codes. Some of the analysis steps were based on the Microstate toolbox (MST, version 1.0) (Poulsen et al., 2018) and Microstate^2^ (version 1.2). For each preprocessed EEG data, the global field power (GFP) was calculated as the standard deviation of the amplitude across all channels at each time point, which reflects the strength of neuronal activity. The local maximal values (peaks) of GFP were extracted, and GFP peaks with the lowest 15% and greater than three times the standard deviation were discarded (Mishra et al., 2020). EEG maps at the surviving GFP peaks (also called original maps) were used for further analysis. There is currently no agreement on how to determine the optimal number of microstate classes due to the different datasets used, and the fact that microstate analysis usually does not consider the polarity may make many clustering number selection criteria inapplicable (Poulsen et al., 2018). Nonetheless, in clinical research, four canonical microstate templates have been highly replicated and well-studied in resting-state EEG (Michel and Koenig, 2018; da Cruz et al., 2020; Gui et al., 2020; Liu et al., 2020). In line with the previous studies, the number of clusters was fixed at four. For each recording, a modified k-means clustering algorithm was utilized to find four templates (cluster centroids; dimensions: 59 × 4) and ignore the polarity (Murray et al., 2008; Poulsen et al., 2018). To avoid systematic variance derived from different group-specific microstate templates, templates of all recordings of patients with DOC were collected and sent to the second modified *k*-means (Van de Ville et al., 2010; Nishida et al., 2013) to get the final four microstate templates. The four microstate templates in our study (Figure 1) were labeled A, B, C, and D, respectively, according to their similarities to the previously reported microstate templates (Michel and Koenig, 2018; da Cruz et al., 2020; Xu et al., 2020). The templates were fitted back to each EEG recording to derive time series sequences of microstates. Then, temporal smoothing was employed in the microstate sequence until no microstate segment was shorter than 30 ms, as previously described (Poulsen et al., 2018; Liu et al., 2020).

For each EEG recording, eight types of microstate features were computed: global explained variance (GEV), mean duration, occurrence, coverage, mean interval, mean GFP, spatial correlation metric (SC), and transition probability (TP). The mean interval and SC are two new features introduced in this study. The mean interval for microstate *X* is calculated using the following equation:


$$MeanInterval_{X}=\frac{1}{N-1}\sum_{i=1}^{N-1}\left(t_{s}^{i+1}-t_{e}^{i}\right),$$

where *N* is the number of microstate segments of microstate *X*; *t_*s*_s* are the start time of microstate *X* segments, and *t_*e*_s* are the end time of microstate *X* segments. SC is the mean absolute correlation values of each microstate template with the maps of a given microstate class. For example, SC_*AB*_ is the average of the absolute correlation coefficients between the template of microstate A and all the maps belonging to microstate B. The first and last microstates of each continuous EEG segment were potentially truncated, and these microstates were not considered when calculating microstate features. Definitions of the microstate features are summarized in Supplementary Table 3. See the references (Murray et al., 2008; Nishida et al., 2013; Wang et al., 2021) for more details on how these features were computed, and see the references (Murray et al., 2008; Nishida et al., 2013; Khanna et al., 2015; Michel and Koenig, 2018) for more details of the neurophysiological interpretation of these microstate features.

### Statistical Analysis

Due to the small sample size, permutation-based non-parametric tests were used for all the statistical tests. In this work, all statistical tests were two-sided, and the significance level is 0.05.

#### For Permutation Tests on Spectral and Microstate Features

For the between-subjects design (e.g., patients at T0 or T1 vs. controls and subgroups at T0 or T1 vs. controls), the condition labels were randomly shuffled. For the within-subjects design (e.g., patients at T1 vs. T0 and each subgroup at T1 vs. T0), the signs of differences between the features of the two conditions (i.e., T1 - T0) were randomly assigned. For each statistical test, all possible permutations were run with the standard error-weighted mean difference (*t*-value) as the statistic. In addition, for the correlation analysis (features vs. CRS-R scores), the orders of the CRS-R scores were randomly shuffled. Similarly, all possible permutations were performed for each correlation analysis with the Spearman correlation coefficient as the statistic. Non-corrected *p*-values were reported due to the limited sample size. For within-subjects subgroup comparisons, there are 16 (i.e., 2^4^) possible permutations in this study. We added one to both the numerator and the denominator to avoid zero when calculating the *p*-values. Therefore, the minimum obtainable *p*-value for within-subjects subgroup comparison was 0.059 (1/17), which was unattainable below the significance level of 0.05.

#### For Permutation Tests on Functional Connectivity

As there were 59 available data channels, there are 1,711 connections in the connectivity network. The number of comparisons involved in the statistical tests was enormous. In this study, a method based on network-based statistic (NBS) was developed to control the family-wise error rates (Zalesky et al., 2010). Similar to the cluster-based permutation test used in EEG time-frequency and continuous signals (Sassenhagen and Draschkow, 2019; Abramov and Miranda de Sa, 2021), determining a *p*-value for each cluster, in this NBS-based method, each connected component was given a *p*-value by the permutation procedure. The *p*-value and sign maps (dimensions: 59 × 59) were generated after all univariate tests. For univariate comparisons, Wilcoxon rank-sum test and Wilcoxon signed-rank test were performed for between-subjects and within-subjects comparisons, respectively. For the correlation analysis, the *p*-value map was created based on the Spearman correlation. The element of the sign map is 1 or −1. For X versus Y, the element is 1 when the median of X is greater than the median of Y for the between-subjects design or the median of X – Y is greater than zero for the within-subjects design or the correlation is positive for correlation analysis. Two significant maps (positive and negative) were created by applying the mask (binarization of the *p*-value map; threshold: 0.05) to the sign map. The connected components were obtained from the significant maps using the MATLAB function *conncomp*. To get the null distributions, the random shuffling procedure was the same as that for the spectral and microstate features mentioned above, and the size of the largest positive and largest negative component was picked and saved for each iteration. The cluster size of a connected component is the sum of the elements of the significant map belonging to the connected component. Here, considering the stability of the results and time consumption, 2,000 permutations were performed if the number of all possible permutations was larger than 2,000. The *p*-value for each observed connected component utilizes the following formula to avoid a zero value:


$$p=2\times \frac{sum\left(abs\left({\text{S}}_{\mathrm{null}}\right)>abs\left(s_{obs}\right)\right)+1}{N+1},$$

where *N* is the number of permutations; *s*_*obs*_ is the size of the observed connected component; and ***S_null_*** is one null distribution selected according to the sign of the size of the observed connected component.

### Classification and Prediction

To examine whether the brain states of patients at T0 and T1 could be characterized by EEG features, SVM, a widely used method in neuroscience (Arbabshirani et al., 2017), was used to build the classification model. To reduce the risk of overfitting, only features with the smallest *p*-values were selected. Furthermore, only the first two principal components of the selected features were retained using principal component analysis (PCA) for further dimensional reduction. SVR was used to predict the CRS-R score at T_*end*_ by EEG features. Correlation-based feature selection is simple to understand (Song et al., 2018), and the feature(s) most significantly correlated with the CRS-R at T_*end*_ was chosen. If the number of selected features (s) was greater than one, the first principal component was retained. The linear SVM and SVR models were conducted with the Python package *scikit-learn*. To avoid an optimistically biased evaluation of the model performance (Scheinost et al., 2019), the nested leave-one-out cross-validation (nested LOOCV) was used to evaluate the tuned SVM and SVR models. For each iteration of the outer LOOCV loop, one subject was split as the test dataset and the remaining subjects as the training dataset. In the inner LOOCV loop, select the best hyperparameters based on grid search hyperparameter optimization and refit a model with the entire training dataset with the determined parameters. This model then was used to predict the test dataset of the outer LOOCV loop. We made the *scikit-learn pipeline* of the preprocessing steps to prevent data leakage in the cross-validation and hyper-parameter tuning procedures.

### Data Visualization

Schemaball plots were made with some modifications to the schemaball project^3^. Networks with EEG electrodes as nodes (Koessler et al., 2009) were created by BrainNet Viewer software (Xia et al., 2013). This is only for a more intuitive representation of connections at the sensor level, and we do not make any interpretations beyond the sensor level. Other plots were generated using OriginPro 2022 Beta2 (OriginLab Corporation, Northampton, MA, United States).

## Results

All eight patients with DOC did not show any clinical improvement after zolpidem administration. Specifically, Subject 1 (I1_T_MSC+), Subject 2 (I2_A_VS), Subject 7 (N3_S_MSC−), and Subject 8 (N4_T_VS) showed drowsiness after zolpidem. The other patients showed no noticeable changes in status based on visual observation. Therefore, all the patients with DOC in this study were zolpidem non-responders.

### Effects of Zolpidem Administration on the Spectral Features

The global relative PSD decreased in the theta band and increased in the beta band in patients with DOC at T1 compared to T0. More precisely, it was significantly reduced in the range of 4.5–7.0 Hz (frequency resolution: 0.5 Hz) and showed no significant differences at other frequencies. Moreover, there was a peak in the global relative PSD for each patient in the theta band at T0, little or more, and the peak shifted toward a higher frequency at T1. To reduce the number of comparisons, the band-level results are reported in the following sections.

**FIGURE 2:**
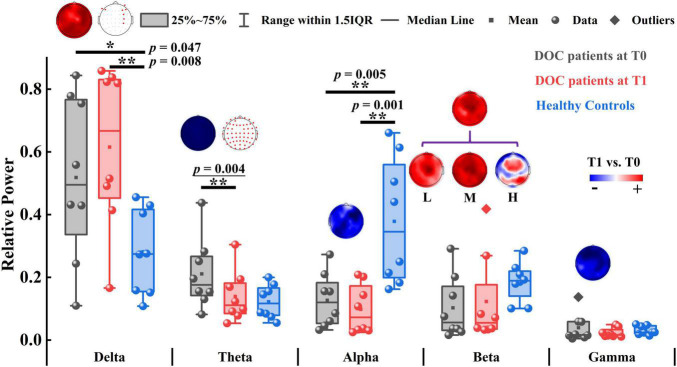
The relative power of patients with DOC and healthy controls. The topographic map above the box plot shows the change in relative power at the channel level before and after zolpidem administration, and, if there is a significant difference, it is marked with a red dot in the other topographic map. Red and blue colors indicate higher and lower relative power for patients at T1 versus patients at T0, respectively. L, M, and H denote the low β-band, middle β-band, and high β-band, respectively. *p < 0.05, **p < 0.01; *p*-value with an underline indicates the minimum obtainable *p*-value is achieved.

We first compared the relative power of each band between T0 and T1. As illustrated in Figure 2, only the global relative theta power showed a significant reduction (*p* = 0.004) at the group level after zolpidem administration. The average decline was 7.20% across all the patients. Notably, this decreasing pattern in the theta band was consistently observed in all the patients (Supplementary Figure 2), although there was large heterogeneity in the patients. Furthermore, the decreasing pattern in the theta band was significant at almost all the electrodes. The alpha and gamma bands showed a similar pattern of change after zolpidem but to a lesser degree (all *ps* > 0.05). In contrast, the global relative power in the delta and beta bands increased after zolpidem administration. After zolpidem, the relative delta power widespread increased at most electrodes and significantly increased in the lateral frontal and right central regions, while the relative beta power (all *ps* > 0.05) also widely increased, but three subbands showed different patterns of change: widespread increase in most electrodes in the low and middle beta bands, and the increase in the frontal and parietal regions in the high beta band.

Next, we tested the differences between the patients and the controls. Briefly, the patients at T0 had stronger relative power in lower frequency bands (delta and theta) and weaker relative power in higher frequency bands (alpha, beta, and gamma). There were significant differences in delta (*p* = 0.047), alpha (*p* = 0.005), and middle beta (*p* = 0.022) bands between the patients at T0 and controls. At T1, there were also significant differences in the delta (*p* = 0.008) and alpha (*p* = 0.001) bands between the patients and the controls. Group average statistics of the patients and the controls are provided in Supplementary Table 4. The complete comparison results of the relative power are summarized in Supplementary Table 5. As for the brain symmetry index, the patients at T0 had increased pdBSI and rBSI in all frequency bands compared to controls. After zolpidem administration, pdBSI and rBSI were reduced in the delta and theta bands, but not significantly (all *p*s > 0.05), see Supplementary Figure 3 and Supplementary Tables 6, 7 for details.

### Effects of Zolpidem Administration on the Functional Connectivity

**FIGURE 3:**
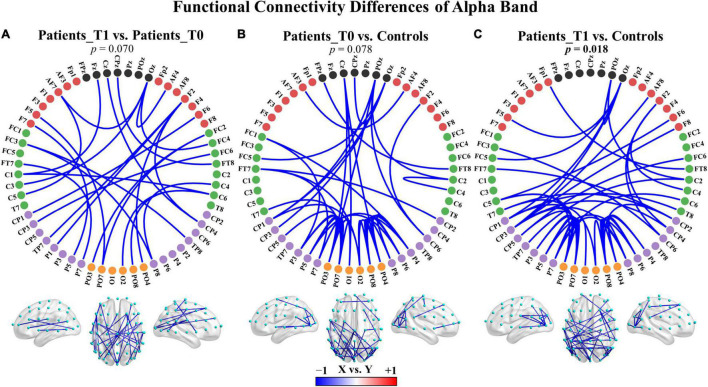
Group differences in functional connectivity of the alpha band. **(A)** Patients at T1 vs. patients at T0. **(B)** Patients at T0 vs. controls. **(C)** Patients at T1 vs. controls. The depth of color indicates the size of the connectivity difference. For *X* vs. *Y*, the blue color indicates *X* < *Y*; the red color indicates *X* > *Y*. For each comparison, only the top 50 connections with the smallest observed *p*-values in the connected component with the smallest *p*-value are displayed. The complete connections of the connected components are provided in Supplementary Figure 5. Bolded *p*-values indicate significant differences.

To examine whether the functional connectivity in the five traditional frequency bands differed between two groups, an NBS-based approach was used to assign a *p*-value to each connected component (not to each connection). No significant differences were found in any of the five bands between the patients at T1 and the patients at T0. Nevertheless, two main patterns of change after zolpidem administration were shown as follows: (a) the delta (*p* = 0.156) and theta (*p* = 0.202) bands demonstrated increased connectivity in the connected component with the smallest *p*-value, and (b) the alpha (*p* = 0.070) and beta (*p* = 0.358) bands showed decreased connectivity in the connected component with the smallest *p*-value. These connected components were mainly involved intrahemispheric and interhemispheric middle- and long-range connections (Supplementary Figures 4A, 5A, 6). For instance, the connected component with the smallest *p*-value in the alpha band showed large-scale connections that decreased after zolpidem, mainly including right intrahemispheric and interhemispheric connections between the posterior and anterior regions and connections with midline electrodes, as shown in Figure 3A and Supplementary Figure 5A.

Additionally, there were also two main patterns of connectivity differences between the patients and healthy controls: (a) the delta, theta, and gamma bands displayed increased connectivity in both the patients at T0 and T1 compared to healthy controls in the connected component with the smallest *p*-value, and (b) the alpha and beta bands showed decreased connectivity in the connected component with the smallest *p*-value. The comparisons were not significant except in the alpha band for the patients at T1 versus controls (*p* = 0.018), see also Figure 3 and Supplementary Figures 4, 5, 7. The hyperconnectivity in the delta, theta, and gamma bands and the hypoconnectivity in the alpha and beta bands all affected large-scale connections and may reflect pathological enhancement/functional reorganization and connectivity disruptions, respectively. The complete results of comparisons are summarized in Supplementary Table 8.

### Effects of Zolpidem Administration on the Microstate Features

**FIGURE 4:**
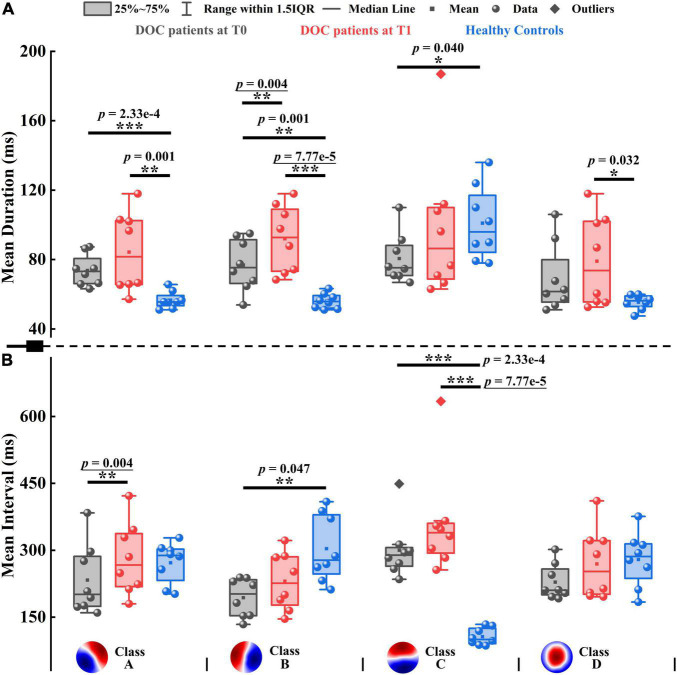
Mean duration and mean interval of patients with DOC and healthy controls. **(A)** Mean duration. **(B)** Mean interval. Four microstate templates of patients with DOC are shown at the bottom of the figure. *p < 0.05, **p < 0.01, ***p < 0.001; *p*-value with an underline indicates the minimum obtainable *p*-value is achieved.

In addition to the results based on the static features, we further explored the spatiotemporal dynamics and investigated how the brain features evolved in the time before and after zolpidem administration and how the brain dynamics were perturbed by DOC. Consistent with previous clinical publications (Zappasodi et al., 2017; da Cruz et al., 2020; Gui et al., 2020), the number of microstate classes was set to four. The microstate templates of the patients are presented in Figure 4 and were labeled A to D, which correspond well with previous studies (Diaz Hernandez et al., 2016; Michel and Koenig, 2018; Comsa et al., 2019; Croce et al., 2020; Xu et al., 2020).

To evaluate the pattern of alterations after zolpidem administration, we examined whether microstate features differed significantly between the patients at T0 and T1. After zolpidem administration, the mean duration of Microstate B and mean interval of Microstate A were both significantly increased (*p* = 0.004), see Figure 4. More specifically, the mean duration of Microstate B and the mean interval of Microstate A increased by an average of 15.150 and 36.625 ms, respectively. Note that the minimum obtainable *p*-value was 0.004 for comparisons between the patients at T0 and T1. Importantly, the pattern of change was the same across all the subjects for the above two features (Supplementary Figure 8). Additionally, the occurrence of Microstate A significantly reduced (*p* = 0.012) after zolpidem administration. No significant differences were found in other microstate features between the patients at T0 and T1. Nevertheless, it is worth noting the tendency of the change of microstate dynamics. When the patients were at T1, at the group level, all microstate classes exhibited longer duration and interval and occurred less frequently than when the patients were at T0. Moreover, Microstate B was of greater mean GFP, explained more variance, and occupied more time after zolpidem administration. In contrast, the other three microstate classes exhibited the opposite pattern. See also Supplementary Tables 9, 10 for more details of the group average statistics and the results of comparisons between groups.

Next, we examined the differences between the patients and healthy controls to explore how neural activity patterns were altered by brain injury. For the healthy subjects, Microstate C was predominant in GEV, mean duration, occurrence, coverage, and mean GFP, with the smallest mean interval compared to the other three microstate classes. For instance, GEV ranged from 5.206 to 31.150% on average in healthy controls, and only GEV of Microstate C (31.150%) was larger than 10%. However, the clear patterns described above disappeared in patients with DOC, see Supplementary Table 9 and Figure 4. It may be caused by the opposite direction of change between Microstate C and the other microstate classes in the patients compared to healthy controls. For example, Microstate C had shorter duration, but Microstates A, B, and D showed longer durations in the patients at T0 compared to controls. In most comparisons, the differences between the patients and healthy controls in Microstates A, B, and C were significant (Supplementary Table 10). Compared to healthy controls, the spatial correlation metrics SC_*AA*_, SC_*BB*_, SC_*CC*_, and SC_*DD*_ were significantly higher in the patients with DOC (Supplementary Figure 9A). The results suggest that the maps belonging to a particular microstate class are with lower variability and are more similar to its template in patients with DOC relative to healthy controls. In addition, the SC_*AB*_, SC_*BA*_, and SC_*BC*_ also significantly increased in the patients. The TPs from Microstates A, B, and D to Microstate C were significantly decreased, while TPs from Microstate C to the other three microstate classes were no significant differences in the patients relative to healthy controls. Conversely, TP_*AB*_, TP_*BA*_, TP_*AD*_, and TP_*DA*_ were significantly higher in the patients compared to controls (Supplementary Figure 9B). Overall, after brain injury, the dynamics of Microstate C were severely affected, and microstates were interconnected and interacted with each other to achieve rebalancing.

We further explored the dynamic changes in EEG features along with the three time points: T0, T1, and T2 (1 h after zolpidem administration to the end time of EEG acquisition). As illustrated in Supplementary Figure 10, all these features displayed a Λ-shape/V-shape behavior from T0 to T2. These results suggest that the EEG response to zolpidem appears to be most pronounced within 1 h.

### Comparisons Between Subgroups With Improvement and With Non-improvement

The eight subjects were divided into two subgroups based on the improvement between the 6-month follow-up and baseline (before zolpidem administration) outcomes: the subgroup with improvement (Subgroup_I), and the subgroup with non-improvement (Subgroup_N), as shown in Table 1. The theoretical minimum obtainable *p*-value for the comparison of subgroups within subjects was 0.059, and that for subgroups between subjects was 0.014. As seen in Supplementary Figure 11, for relative power, Subgroup_I showed a reduction in the theta (*p* = 0.059) and alpha (*p* = 0.059) bands after zolpidem, while Subgroup_N also showed a reduction in the theta band (*p* = 0.059), but to a lesser degree. On the other hand, Subgroup_I tended to have greater rBSI and mean duration than Subgroup_N. There were significant differences in the theta band at T0 (*p* = 0.042) and the gamma band at T1 (*p* = 0.014) between the two subgroups in rBSI. In addition, Subgroup_I had stronger connectivity in the connected component with the smallest *p*-value, mainly involving long-range connections, in the alpha band at T0 (*p* = 0.169) and the theta band at T1 (*p* = 0.056), see Supplementary Figure 12. The mean functional connectivity strength of the connected component (FCSCC) was computed for both subgroups. At T0, Subgroup_I had a significantly greater mean FCSCC in the alpha band (*p* = 0.014) compared to Subgroup_N. Moreover, Subgoup_I exhibited a large increase in the mean duration of Microstates B (*p* = 0.059) and D (*p* = 0.059), and a large decrease in the occurrence of Microstates A (*p* = 0.059) and C (*p* = 0.059) after zolpidem, whereas there was only a noticeable increase in the mean duration of Microstate B (*p* = 0.059) in Subgroup N (Supplementary Figure 13). In summary, Subgroup_I had stronger asymmetry of brain signals, greater connectivity in the theta-alpha band at T0, and tended to have greater changes in EEG features compared to Subgroup_N. The complete results are presented in Supplementary Tables 5–8, 10.

### EEG Features Discriminate the Different Brain States at T0 and T1

**FIGURE 5:**
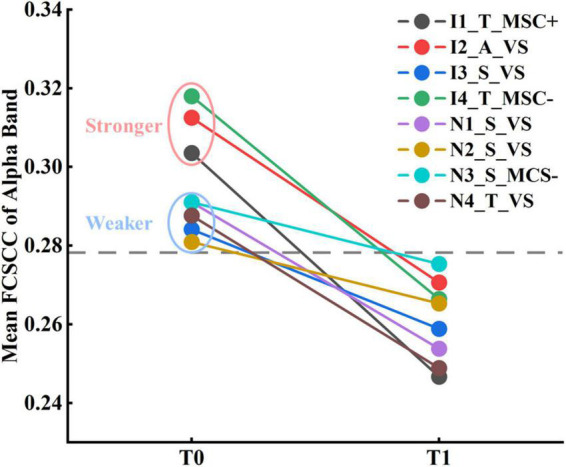
Results of classification between patients at T0 and T1 by the mean FCSCC of the alpha band.

Although none of the eight subjects were aroused by zolpidem, it remains unknown whether the two states of the patients before and after zolpidem administration could be characterized by EEG features. Linear kernel SVM models were conducted to classify the two brain states. Only the features with the smallest *p*-value (*p* = 0.004) were selected. For functional connectivity, the connected components with the smallest *p*-value of the delta and alpha bands were used to compute the mean FCSCCs to reduce the feature dimensions. Mean FCSCC significantly increased in the delta band (*p* = 0.004) and decreased in the alpha band (*p* = 0.004). Therefore, the relative power in the theta band, the mean duration of Microstate B, the mean interval of Microstate A, and the mean FCSCCs of the delta and alpha bands were selected for the linear SVM model. Given the relatively small sample size and the possible feature redundancy and noise, we used PCA for further dimensionality reduction, keeping only the first two principal components. In addition, we utilized nested LOOCV to overcome the optimistically biased performance. The *scikit-learnpipeline* method was used to prevent potential data leakage during preprocessing (scaling features and PCA). The area under the receiver operating characteristic (ROC) curve was one. The results indicate that the brain states before and after zolpidem administration could be distinguished using only EEG features for each patient, although they were all zolpidem non-responders. In a deeper observation of the selected EEG features, we found that the mean FCSCC of the alpha band alone could completely characterize the different brain states at T0 and T1, as shown in Figure 5. Moreover, three subjects in Subgroup_I showed stronger connectivity at T0 compared to the other patients. The performance of the SVM model without PCA is listed in Supplementary Table 11.

### EEG Features Predict the Long-Term Prognostic Outcome

**FIGURE 6:**
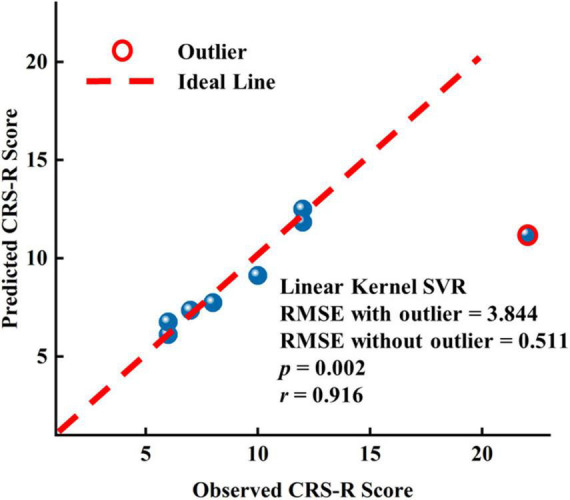
Results of the linear kernel SVR model to predict the long-term prognostic outcome by EEG features at T0 and T1.

To assess the predictive capability of baseline EEG features for long-range CRS-R scores in the patients with DOC, we constructed three linear kernel SVR models using EEG features only before or after zolpidem administration and both, respectively. We also utilized the nested LOOCV procedure, as used in SVM. The prediction performance was evaluated using the root-mean-square error (RMSE). Since there were only eight samples, we selected one feature with the smallest *p*-values for the first two models, respectively, and two features used in the first two models were performed PCA, and then only the first principal component was used in the third model. The feature selected for the first model was the mean FCSCC of the alpha band at T0 (*p* = 2.480 e-5), and for the second model was the mean FCSCC of the theta band at T1 (*p* = 0.002). The connection components used to calculate the mean FCSCCs are provided in Supplementary Figure 14. The above two connected components with a positive correlation between connectivity and the CRS-R score mostly involved interhemispheric connections between the anterior and posterior areas. It also indicated that patients with more preserved interhemispheric connections may have better long-term outcomes. The third SVR model, using the features of the two time points, exhibited the best performance (RMSE without the outlier = 0.511), as shown in Figure 6. The correlation coefficient between the actual CRS-R scores and predicted CRS-R scores was 0.916 (*p* = 0.002). The results of correlation analysis are presented in Supplementary Tables 12–14. See also Supplementary Table 15 for the results of other SVR models.

## Discussion

To the best of our knowledge, our study represents the most comprehensive research to date using EEG features to explore the effects of zolpidem in patients with DOC and the potential clinical implications of zolpidem and EEG for three reasons. First, multiple EEG analysis methods were used to uncover zolpidem-induced brain changes from multiple perspectives, including using static and dynamic features. Second, our results are based on the multilevel and multiband analysis of patients with DOC: at the group, subgroup, and individual levels; global and channel levels; and in five canonical frequency bands. Third, the potential of EEG features as biomarkers and for classification and prediction was explored in this work. Despite the substantial individual heterogeneity in the patients with DOC, we demonstrated that the patients manifested a consistent pattern of changes in some EEG features (e.g., relative power in the theta band and duration of Microstate B) after zolpidem administration, accompanied by apparent individual differences in expression levels. Furthermore, brain states before and after zolpidem administration can be identified by the one-dimensional feature alone. Finally, we found that the functional connectivity at the baseline, both before and after zolpidem administration, was a potential indicator for predicting a long-term prognosis at the single-subject level.

### Changes in EEG Features After Zolpidem Administration

Two lines of evidence from our work converge on the conclusion that all patients with DOC, albeit all zolpidem non-responders and the large heterogeneity of them, showed consistent patterns of change in certain EEG features after zolpidem administration. First, for static features, all the patients showed a reduction in relative theta power and decreased strength of connectivity of the alpha band after zolpidem administration. Second, for temporal dynamics, alterations in the mean duration of Microstate B and the mean interval of Microstate A were significant, and all the patients showed an increased pattern. These results provide key insights into how zolpidem alternates brain function in patients with DOC from a comprehensive perspective.

#### Static Features

We also found that global relative theta-alpha power decreased at the group level after zolpidem administration. In addition, the spectrum shifted toward a higher frequency at T1. These findings are in line with the previous studies using EEG and MEG for an individual-based analysis of patients with severe brain injury (Hall et al., 2010; Williams et al., 2013; Arnts et al., 2020; Sripad et al., 2020). Two MEG studies showed a clear reduction in power after zolpidem for the frequency range of 4–10 Hz within the periinfarct region (Hall et al., 2010) and 4–12 Hz (Sripad et al., 2020), respectively. Williams et al. (2013) demonstrated that EEG power in the range of ∼6–10 Hz was sharply reduced in all three patients with severe brain injury, and the peaks in power in the range of 6–12 Hz produced shifts toward higher frequencies. Arnts et al. (2020) reported similar patterns: EEG theta-alpha power decreased and an overall shift toward higher frequencies of the spectrum. It is worth noting that the power reduction in the theta-alpha band was nearly distributed throughout the whole brain (Williams et al., 2013; Arnts et al., 2020; Sripad et al., 2020). On the other hand, the results of global relative beta power were inconsistent across published studies on zolpidem responders. Some authors reported an increased pattern (Williams et al., 2013; Arnts et al., 2020; Sripad et al., 2020), while others showed the opposite (Hall et al., 2010). For instance, Hall et al. (2010) reported that MEG beta (15–30 Hz) power reduced within the periinfarct region after zolpidem uptake. A small increase in the relative beta power of EEG, mainly over frontal, temporal, and central/parietal regions after zolpidem administration was reported by Arnts et al. (2020). In our study, the increased pattern in the high-beta band was mainly in the central frontal and parietal regions. Therefore, one explanation for this discrepancy is that the pattern of change in the beta power differs in different beta subbands and brain regions. Normally, beta oscillations are most evident frontally, and frontal beta oscillations are attributed to different brain functions (e.g., consciousness, attention, and executive control of movement) (Schmidt et al., 2019; Vernet et al., 2019). Moreover, a previous study suggested that increased high-beta activity seems a prerequisite for behavior to occur under sedation (van Lier et al., 2004). Patients with DOC share some common pathophysiological mechanisms (Giacino et al., 2014) and also have complex individual discrepancies. Thus, it is not surprising that patients show different changes in some features. The results for a single individual cannot be over-explained. Further studies with larger sample sizes might provide stronger evidence on how the beta power alters after zolpidem. Our study showed that the relative delta power increased at most electrodes at the group level. Although alterations in the delta band after intake of zolpidem were not clearly described in studies of zolpidem responders; it was visible from the results in the literature (e.g., spectrograms) that the delta power of at least some channels was elevated (Williams et al., 2013; Arnts et al., 2020; Sripad et al., 2020). Given the complex influence of different high-pass filters on the delta power, it is difficult to directly compare the results across studies. Moreover, the patients in the studies (Hall et al., 2010; Williams et al., 2013; Arnts et al., 2020) were all zolpidem responders and had different etiologies and disease severity. Importantly, they were with a long-time post-injury (2–8 years, only according to the obtainable data) before exposure to zolpidem. The patient in the study (Sripad et al., 2020) was fully conscious, previously a zolpidem responder, but exhibited no clinical effects after zolpidem at the time of measurement. Correspondingly, all the patients in our study showed no clinical improvement, and the average time from the brain injury of the patients was 4.5 months (range = 3–12 months). Surprisingly, the main power alterations described above (e.g., decrease in the theta/alpha band and increase in the delta/gamma band) also hold in healthy populations (Patat et al., 1994; Landolt et al., 2000). The decreased theta power was the most consistent result across studies. Together, these findings appear to provide evidence that the power alterations after zolpidem administration are neither zolpidem responder-specific changes nor the result of behaviourally introduced performance confounding but may simply be a brain reaction to zolpidem regardless of the conditions the subject is in. Based on the current evidence, the results in terms of power do not serve as a neurological marker for zolpidem responders. Given that previous studies in patients with brain injury have largely focused on zolpidem responders, it is crucial to point this out to avoid the overinterpretation of power changes.

Additionally, we found decreased connectivity (i.e., PLV) in the theta-alpha band and increased connectivity in the delta/beta band in the connected components with the smallest *p*-value for T1 versus T0. The size of the connected component with the smallest *p*-value in delta/theta/beta was small. Large-scale disruptions of long-range connections in the alpha band and the mean FCSCC were consistently reduced in all the patients. It was shown high EEG spatial coherence was sharply reduced in the range of ∼6–10 Hz in all three patients with severe brain injury (Williams et al., 2013). Arnts et al. (2020) reported decreased MEG beta band connectivity (cAEC) throughout the brain after zolpidem administration in a patient with hypoxic-ischemic brain injury. Conversely, Sripad et al. (2020) showed stronger MEG connectivity (PLI) between the left frontal and temporal brain regions in the beta-gamma band after zolpidem intake. Due to the very small sample size and wide variations in the methods of constructing connectivity, data modality, etiology of patients (Hall et al., 2010; Williams et al., 2013; Arnts et al., 2020; Sripad et al., 2020), and so on, the results in the literature are too mixed to draw solid conclusions and are difficult to compare or corroborate. Furthermore, phase- and amplitude-coupling patterns may partly reflect different neuronal mechanisms (Siems and Siegel, 2020; Duclos et al., 2021). In general, the presence of high-frequency oscillations, corticocortical and thalamocortical connections (structural and functional) is associated with higher levels of consciousness (Mofakham et al., 2021; Oberto et al., 2022). Delta activity and synchronization appear to be higher when the cortical arousal is at a lower level (Sitt et al., 2014; Frohlich et al., 2021). Previous work has shown that the strength of synchronization of the drowsy state is larger than that of the alert state in the delta band but lower in the alpha band (Chen et al., 2018). Taken together, we speculate that the change in functional connectivity after zolpidem may reflect the decrease in consciousness, and zolpidem may introduce a sedative-like effect in patients with DOC in our study.

#### Microstate Dynamics

Furthermore, we observed, for the first time, alterations of microstate dynamics in patients with DOC after intake of zolpidem and provided us with a new and important perspective. After zolpidem administration, the mean duration and mean interval were increased, and the occurrence was decreased in all four microstate classes at the group level. The alterations in the mean duration of Microstate B and mean interval of Microstate A were significant, and all the patients showed an increased pattern. Prior studies have shown that the temporal dynamics of all microstate classes slow down during drowsiness (Comsa et al., 2019) and after deep anesthesia (Artoni et al., 2021). Microstates A and B may reflect the extrinsic system, while Microstates C and D may reflect the intrinsic system (Giordano et al., 2018). In our study, the microstate temporal dynamics became slower too, which seems to indicate a reduced demand for relatively complex brain functions or weaker support for complex brain activity after zolpidem intake. We speculate that the two aforementioned features (mean duration of Microstate B and mean interval of Microstate A) are indicators of the response of the brain to zolpidem, as well as theta power.

It is worth noting that, whether in the eyes-open or eyes-closed state or even in the deeply anesthetized state, a consistent and striking characteristic of microstates in healthy individuals is the predominance of Microstate C in terms of occurrence and duration, and so on (Michel and Koenig, 2018; Zanesco et al., 2020, 2021; Artoni et al., 2021). Importantly, this dominant pattern of Microstate C was tremendously reduced or even completely disappeared in patients with DOC. On the other hand, Microstates A and B exhibited the opposite pattern to Microstate C. In addition, the transition probabilities from the other three microstate classes to Microstate C were significantly lower, while the transition probabilities between Microstates A and B, and between Microstates A and D were significantly higher in patients with DOC compared to healthy individuals. Furthermore, the spatial correlation of each microstate template with its microstate-specific maps intensified. In the patients with DOC, Gui et al. (2020) reported the mean duration of the L–R map (averaging templates A and B) was higher, and the occurrence of the L-R map was lower than that in healthy controls. Spatial and temporal variability increased with the consciousness levels (Cai et al., 2020). Thus, our study suggests that altered consciousness following brain injury is accompanied by a general disruption in the order and dynamic balance of microstates. The decrease in brain topographic variability and the slowing down of brain state switching appear to indicate a reduction in the complexity of brain network dynamics, reflecting the decline in consciousness.

### The Potential Value of EEG Features/Zolpidem in Patients With Disorders of Consciousness

Herein, we capitalized on various EEG methods to explore the patterns of change in EEG features after zolpidem and the potential clinical value of EEG neural markers and zolpidem. First, our study extends our understanding of the effects of zolpidem on the brain. This was discussed in detail in the previous section. Second, it is possible to use EEG features to assess a brain state and the level of consciousness and even predict long-range outcomes in patients with DOC at the single-patient level. Despite some promising MRI and EEG studies (Engemann et al., 2018; Song et al., 2018), there are still few reliable methods to assist in the diagnosis and prognosis of patients with DOC at the individual level (Giacino et al., 2014; Pauli et al., 2020; Song et al., 2020). Furthermore, the majority of current prognostic models do not directly predict the detailed scores but coarse-grained outcomes (e.g., a favorable versus unfavorable outcome) (Song et al., 2020). In this study, although none of the eight patients with DOC were zolpidem responders, the brain states before and after zolpidem intake could be characterized by the mean FCSCC of the alpha band. It appears that Subgroup_I had a stronger connection in the alpha band before zolpidem intake. The mean FCSCC of the theta band at T1 was also significantly higher in Subgroup_I than that in Subgroup_N. Moreover, Subgroup_I showed greater asymmetry of neural signals compared to Subgroup_N and tended to have a greater response to zolpidem in EEG features after zolpidem. This may be explained by better reconfiguration of brain function during the recovery process, more preservation of brain function, and greater modifiability of brain states in Subgroup_I. For prediction, despite the small sample size, we made accurate predictions of the long-range outcome for each patient. The SVR model using the features both before and after zolpidem exhibited the best performance. In addition, the mean FCSCCs in the theta band after zolpidem and the alpha band before zolpidem were both significantly and positively correlated with the CRS-R scores. Furthermore, the previous study has shown that survivors have stronger alpha-band functional connectivity (PLV) than non-survivors in patients with acute coma (Alnes et al., 2021). Combining the results of our study, we extrapolated that patients retaining stronger functional connectivity in the alpha band before zolpidem and the theta band after zolpidem have a better prognosis. Overall, some EEG features have the potential to serve as useful neural markers to predict the potential for recovery, even at a fine-grained scale. Our study also indicates the additional clinical value of zolpidem beyond the immediate awakening of selected patients with DOC.

Currently, the major mechanism model associated with the loss and recovery of consciousness named the mesocircuit model was proposed by Schiff (2010). As described in this model, the active inhibitory output of the internal globus pallidus (GPi) to the central thalamus is inhibited by zolpidem. Furthermore, the thalamocortical and thalamus-striatum pathways are transiently restored, and then cortical dynamics associated with consciousness increase and consciousness is recovered. However, a recent study has found that the external globus pallidus (GPe) plays an important role in maintaining a state of consciousness (Zheng and Monti, 2019). This study claimed the presence of direct connections between the GPe and the prefrontal cortex/central thalamus involved in the regulation of arousal and cognition. The GPi, on the other hand, is more restricted to cortical and thalamic regions associated with movement. Previous studies have shown that patients with traumatic brain injury have a higher response rate than those with stroke (Zhang et al., 2021), and patients with non-brain-stem injuries are more likely to have functional recovery after zolpidem intake (Du et al., 2014). Furthermore, the time post-injury of DOC in zolpidem responders reported in previous studies was usually longer than 2 years (Clauss and Nel, 2006; Whyte and Myers, 2009; Williams et al., 2013; Arnts et al., 2020; Sripad et al., 2020). The distribution and density of neurotransmitter receptor interact with structural and functional connectivity, and the ionotropic GABA_*A*_ receptors are dominant for the alpha and gamma bands (Hansen et al., 2021). Thus, we presume that, in zolpidem responders, the brain structure at key sites is preserved (possibly going dormant), or there is some important restoration of structure and function with recovery that forms the basis of consciousness reestablishment, accompanied by receptor changes (e.g., the distribution and density).

### Limitations and Future Directions

Several considerations should be taken into account. First, none of the patients with DOC in the study showed improvement after zolpidem administration. Therefore, we were unable to directly compare the EEG features of zolpidem responders and non-responders in this study. Second, given the small sample size and the heterogeneity of patients (e.g., etiology, location of impairment, and treatment), caution is required for the interpretation of the results. Nevertheless, given that most of the previous studies are case reports on zolpidem responders (Hall et al., 2010; Arnts et al., 2020; Sripad et al., 2020), the sample size in this study is larger in this field and provides more reliable results. Third, the heterogeneity of patients with DOC may attenuate the zolpidem-induced effect we observed. For this, we performed a multilevel analysis. Fourth, since features from all eight patients were used in feature selection that may introduce data leakage, the findings need to be confirmed by larger sample size research with a more rigorous procedure. In addition, the results of patients after zolpidem administration were computed using the signals around 20–40 min after zolpidem uptake. If different signal periods are used for analysis, the results may also vary. Moreover, different data modalities (e.g., EEG and MRI) and methods (e.g., different functional connectivity construction approaches) can reflect different neuronal mechanisms (Siems and Siegel, 2020; Duclos et al., 2021), and comparisons of studies need to be very careful.

To explore the mechanism of action and potential value of zolpidem, given its low response rate, international collaboration is needed to construct datasets of various etiologies and different states of consciousness. Meanwhile, owing to the low spatial resolution of EEG, MRI data are also needed for a better exploration of mechanisms. In addition, source space EEG analysis in patients with brain injury is a challenging but fascinating topic. Moreover, more rigorous experiments are needed to investigate the relationship between the changes in EEG features and the level of consciousness and to uncover what neural markers can indicate or reflect the dramatic changes in zolpidem responders. Finally, in the future, we will investigate the robustness of the prediction model using EEG features in a large dataset of patients with DOC.

## Conclusion

In a nutshell, we capitalized on static and dynamic EEG features to fully uncover zolpidem-induced alterations in eight patients with DOC and predict long-term outcomes at the single-subject level. Our results show that patients with DOC, albeit non-responders to zolpidem, present similar patterns of change compared to responders in certain EEG features, e.g., in the relative theta power. Surprisingly, despite the heterogeneity of the patients, they showed the consistent change direction in several EEG features like relative theta power, mean duration Microstate B, and alpha band functional connectivity, extending the previous studies. Furthermore, the brain states before and after zolpidem intake can be completely characterized, and long-range outcomes of patients with DOC can be predicted well at the single-patient level by functional connectivity. These results extend our understanding of the effects of zolpidem on the brain and open avenues for potential clinical applications of EEG and zolpidem.

## Data Availability Statement

The EEG datasets used in this article are not readily available because of regulations and the sensitive nature of the clinical data. EEG features used in this study are available from the corresponding authors upon reasonable request. Detailed statistical results and data files of the microstate templates are provided in the Supplementary Material.

## Ethics Statement

The studies involving human participants were reviewed and approved by the Ethics Committee of the PLA General Hospital. The patients/participants provided their written informed consent to participate in this study.

## Author Contributions

ZH: conceptualization, methodology, software, formal analysis, validation, visualization, interpretation of data, writing the original draft, review, and editing. XX: project administration, resources, conceptualization, data curation, supervision, interpretation of data, review, and editing. YB and YW: investigation, data curation, review, and editing. WD: funding acquisition, project administration, supervision, review, and editing. All authors contributed to the article and approved the submitted version.

## Conflict of Interest

The authors declare that the research was conducted in the absence of any commercial or financial relationships that could be construed as a potential conflict of interest.

## Publisher’s Note

All claims expressed in this article are solely those of the authors and do not necessarily represent those of their affiliated organizations, or those of the publisher, the editors and the reviewers. Any product that may be evaluated in this article, or claim that may be made by its manufacturer, is not guaranteed or endorsed by the publisher.

## Footnotes

1
http://sccn.ucsd.edu/

2
https://www.thomaskoenig.ch/index.php/software/microstates-in-eeglab/getting-started

3
https://github.com/okomarov/schemaball
