# Supplementary material for: EEG Evidence Reveals Zolpidem-Related Alterations and Prognostic Value in Disorders of Consciousness
Source: Front Neurosci. 2022 Apr 27;16:863016. doi: 10.3389/fnins.2022.863016 (PMC9093050; doi:10.3389/fnins.2022.863016)
Supplement: Supplementary file 1 [file Data_Sheet_1.pdf]

# *Supplementary Material*

## 1 EEG data acquisition of healthy controls

Eyes-closed resting-state EEG data were acquired from a 64-channel electrode cap (NeuSen W64, Neuracle, China, http://www.neuracle.cn/productinfo/148706.html), with 59 scalp electrodes (Ag/AgCl) placed according to 10-10 international system. EEG data were recorded with a Neuracle amplifier with 24-bit resolution, a sampling rate of 1000 Hz, and lowpass filtered with cutoff frequency (−3 dB) at 250 Hz for between approximately 3 to 5 minutes. The reference electrode was located at electrode CPz and the ground electrode was located at AFz. During recording, the impedance was kept below 10 kΩ for all scalp electrodes. EEG data were recorded in a specific, dimly lit, and sound-attenuated room.

## 2 Supplementary Tables

### Supplementary Table 1. Demographics of healthy controls.

| ID | Gender | Age (years) | Handedness |
|---|---|---|---|
| 1 | Male | 58 | Right |
| 2 | Male | 41 | Right |
| 3 | Female | 60 | Left |
| 4 | Male | 53 | Right |
| 5 | Male | 40 | Right |
| 6 | Male | 32 | Right |
| 7 | Male | 50 | Right |
| 8 | Male | 48 | Right |

### Supplementary Table 2. Statistics of EEG preprocessing metrics for patients and controls.

| Parameters | Patients_T0 | Patients_T1 | Controls |
|---|---|---|---|
| Removed channels (n) | 0.4 ± 0.5<br>0–1 | 0.4 ± 0.5<br>0–1 | 0.6 ± 1.0<br>0–3 |
| Rejected components (n) | 22.8 ± 5.1<br>12–30 | 25.1 ± 5.0<br>17–32 | 11.1 ± 4.2<br>4–16 |
| Length of EEG retained (seconds) | 591.8 ± 27.6<br>566.3–658.6 | 1130.1 ± 71.7<br>989.0–1194.2 | 238.5 ± 52.7<br>179.1–301.4 |

Mean ± standard deviation and range for each parameter are provided in the table.

### Supplementary Table 3. The definitions of the microstate features.

| Microstate feature | Definition |
|---|---|
| GEV | The percentage of topographic variance explained by each microstate class. |
| Mean duration | The average of the continuous length of time during which the EEG time series is determined to be a certain microstate class. |
| Occurrence | The average number of occurrences per second of each microstate class. |
| Coverage | The percentage of total analysis time occupied by each microstate class. |
| Mean interval | The average time across all the intervals from the end of a particular microstate class to the start of the next same microstate class. |
| Mean GFP | The average amplitude of GFP during each microstate class dominance. |
| Spatial correlation metric | The mean absolute correlation values of each microstate template with the maps of a given microstate class. |
| Transition probability | The probability (observed transition probability minus expected transition probability) from each microstate class to another. |

## Supplementary Table 4. Group average statistics (±*SD*) of the Patients at T0, Patients at T1, and Healthy Controls for the computed relative power, pdBSI, and rBSI in five frequency bands.

| | Group | $\delta$ | $\theta$ | $\alpha$ | $\beta$ | $\gamma$ |
|---|---|---|---|---|---|---|
| Relative power | Patients_T0 | 0.519 ± 0.264 | 0.211 ± 0.112 | 0.127 ± 0.086 | 0.103 ± 0.100 | 0.039 ± 0.045 |
| | Patients_T1 | 0.615 ± 0.256 | 0.139 ± 0.081 | 0.099 ± 0.078 | 0.123 ± 0.143 | 0.023 ± 0.015 |
| | Controls | 0.282 ± 0.136 | 0.123 ± 0.054 | 0.378 ± 0.201 | 0.185 ± 0.061 | 0.031 ± 0.014 |
| pdBSI | Patients_T0 | 0.169 ± 0.052 | 0.198 ± 0.085 | 0.186 ± 0.075 | 0.179 ± 0.060 | 0.184 ± 0.039 |
| | Patients_T1 | 0.134 ± 0.067 | 0.166 ± 0.098 | 0.175 ± 0.110 | 0.179 ± 0.082 | 0.186 ± 0.077 |
| | Controls | 0.155 ± 0.049 | 0.134 ± 0.043 | 0.114 ± 0.015 | 0.122 ± 0.035 | 0.145 ± 0.056 |
| rBSI | Patients_T0 | 0.127 ± 0.113 | 0.134 ± 0.098 | 0.105 ± 0.074 | 0.096 ± 0.119 | 0.106 ± 0.085 |
| | Patients_T1 | 0.095 ± 0.093 | 0.126 ± 0.110 | 0.127 ± 0.140 | 0.137 ± 0.110 | 0.100 ± 0.088 |
| | Controls | 0.053 ± 0.032 | 0.062 ± 0.048 | 0.056 ± 0.021 | 0.053 ± 0.030 | 0.059 ± 0.041 |

Mean ± standard deviation for each parameter is provided in the table.

## Supplementary Table 5. Results of analysis of the difference in relative power.

| | $N_{all}$ | $\delta$ | $\theta$ | $\alpha$ | $\beta$ | Low $\beta$ | Middle $\beta$ | High $\beta$ | $\gamma$ |
|---|---|---|---|---|---|---|---|---|---|
| **Patients_T1 vs. Patients_T0** | 256 | 0.082 | **−0.004** | −0.518 | 0.409 | 0.603 | 0.346 | 0.922 | −0.377 |
| Subgroup_I_T1 vs. Subgroup_I_T0 | 16 | 0.176 | −0.059 | −0.059 | −0.765 | −0.529 | −0.765 | 0.765 | −0.647 |
| Subgroup_N_T1 vs. Subgroup_N_T0 | 16 | 0.294 | −0.059 | 0.412 | 0.176 | 0.176 | 0.059 | −0.882 | −0.765 |
| Subgroup_I_T0 vs. Subgroup_N_T0 | 70 | 0.718 | −0.521 | 0.859 | −0.887 | −0.803 | −0.746 | 0.915 | −0.803 |
| Subgroup_I_T1 vs. Subgroup_N_T1 | 70 | 0.352 | −0.127 | −0.239 | −0.437 | −0.408 | −0.465 | 0.972 | 0.859 |
| Subgroup_I vs. Subgroup_N ( Δ ) | 70 | 0.380 | −0.634 | **−0.014** | −0.380 | −0.127 | −0.352 | 0.887 | 0.803 |
| **Patients_T0 vs. Controls** | 12870 | **0.047** | 0.051 | **−0.005** | −0.068 | −0.154 | **−0.022** | −0.235 | 0.736 |
| Subgroup_I_T0 vs. Controls | 495 | **0.040** | 0.127 | **−0.048** | −0.083 | −0.190 | **−0.026** | −0.313 | 0.766 |
| Subgroup_N_T0 vs. Controls | 495 | 0.141 | 0.050 | **−0.036** | −0.171 | −0.373 | −0.095 | −0.302 | 0.724 |
| **Patients_T1 vs. Controls** | 12870 | **0.008** | 0.654 | **−0.001** | −0.283 | −0.406 | −0.335 | −0.287 | −0.284 |
| Subgroup_I_T1 vs. Controls | 495 | **0.006** | −0.393 | **−0.014** | −0.111 | −0.081 | **−0.038** | −0.472 | −0.417 |
| Subgroup_N_T1 vs. Controls | 495 | 0.073 | 0.234 | **−0.046** | −0.651 | −0.972 | −0.831 | −0.226 | −0.335 |

$N_{all}$ is the number of all possible permutations.
The results of the statistical analysis are reported as *p*-values in the table.
For X vs. Y, a negative sign before a *p*-value indicates X < Y.
Bolded *p*-values indicate significant differences.
*p*-value with underline indicates the minimum obtainable *p*-value is achieved.

## Supplementary Table 6. Results of analysis of the difference in pdBSI.

| | $N_{all}$ | $\delta$ | $\theta$ | $\alpha$ | $\beta$ | Low $\beta$ | Middle $\beta$ | High $\beta$ | $\gamma$ |
|---|---|---|---|---|---|---|---|---|---|
| **Patients_T1 vs. Patients_T0** | 256 | −0.058 | −0.198 | −0.642 | 0.992 | −0.961 | 0.735 | −0.860 | 0.930 |
| Subgroup_I_T1 vs. Subgroup_I_T0 | 16 | −0.176 | −0.176 | −0.412 | 0.647 | −0.882 | 0.529 | 0.647 | 0.882 |
| Subgroup_N_T1 vs. Subgroup_N_T0 | 16 | −0.412 | −0.647 | 0.882 | −0.647 | 0.765 | −0.765 | −0.294 | −0.647 |
| Subgroup_I_T0 vs. Subgroup_N_T0 | 70 | 0.211 | 0.127 | 0.127 | 0.437 | 0.268 | 0.408 | 0.521 | 0.239 |
| Subgroup_I_T1 vs. Subgroup_N_T1 | 70 | 0.465 | 0.324 | 0.465 | 0.437 | 0.465 | 0.380 | 0.521 | 0.127 |
| Subgroup_I vs. Subgroup_N ( $\Delta$ ) | 70 | −0.634 | −0.352 | −0.634 | 0.549 | −0.887 | 0.606 | 0.437 | 0.408 |
| **Patients_T0 vs. Controls** | 12870 | 0.588 | 0.081 | **0.017** | **0.029** | **0.014** | 0.064 | **0.036** | 0.125 |
| Subgroup_I_T0 vs. Controls | 495 | 0.228 | **0.014** | **0.002** | **0.022** | **0.010** | **0.032** | **0.046** | 0.103 |
| Subgroup_N_T0 vs. Controls | 495 | −0.706 | 0.599 | 0.222 | 0.131 | 0.103 | 0.254 | 0.149 | 0.512 |
| **Patients_T1 vs. Controls** | 12870 | −0.475 | 0.410 | 0.173 | 0.083 | **0.040** | 0.089 | 0.126 | 0.261 |
| Subgroup_I_T1 vs. Controls | 495 | −0.958 | 0.212 | **0.042** | **0.036** | **0.010** | **0.026** | 0.069 | 0.087 |
| Subgroup_N_T1 vs. Controls | 495 | −0.220 | 0.925 | 0.554 | 0.308 | 0.216 | 0.349 | 0.373 | 0.901 |

$N_{all}$ is the number of all possible permutations.
The results of the statistical analysis are reported as *p*-values in the table.
For X vs. Y, a negative sign before a *p*-value indicates X < Y.
Bolded *p*-values indicate significant differences.
*p*-value with underline indicates the minimum obtainable *p*-value is achieved.

## Supplementary Table 7. Results of analysis of the difference in rBSI.

| | $N_{all}$ | $\delta$ | $\theta$ | $\alpha$ | $\beta$ | Low $\beta$ | Middle $\beta$ | High $\beta$ | $\gamma$ |
|---|---|---|---|---|---|---|---|---|---|
| **Patients_T1 vs. Patients_T0** | 256 | −0.152 | −0.821 | 0.510 | 0.058 | 0.136 | 0.058 | 0.082 | −0.735 |
| Subgroup_I_T1 vs. Subgroup_I_T0 | 16 | −0.294 | −0.647 | 0.647 | 0.176 | 0.059 | 0.176 | 0.176 | 0.412 |
| Subgroup_N_T1 vs. Subgroup_N_T0 | 16 | −0.294 | 0.529 | 0.882 | 0.294 | 0.529 | 0.294 | 0.294 | −0.176 |
| Subgroup_I_T0 vs. Subgroup_N_T0 | 70 | 0.099 | **0.042** | 0.634 | 0.408 | 0.662 | 0.211 | 0.352 | 0.549 |
| Subgroup_I_T1 vs. Subgroup_N_T1 | 70 | **0.127** | 0.408 | 0.521 | 0.296 | 0.437 | 0.296 | 0.324 | **0.014** |
| Subgroup_I vs. Subgroup_N ( $\Delta$ ) | 70 | −0.606 | −0.408 | 0.662 | 0.944 | 0.718 | −0.972 | −0.915 | 0.127 |
| **Patients_T0 vs. Controls** | 12870 | **0.048** | 0.085 | 0.089 | 0.460 | 0.080 | 0.629 | 0.623 | 0.170 |
| Subgroup_I_T0 vs. Controls | 495 | **0.004** | **0.006** | 0.058 | 0.131 | 0.050 | 0.115 | 0.188 | 0.091 |
| Subgroup_N_T0 vs. Controls | 495 | 0.548 | 0.919 | 0.179 | −0.980 | 0.169 | −0.571 | −0.712 | 0.433 |
| **Patients_T1 vs. Controls** | 12870 | 0.271 | 0.137 | 0.237 | **0.028** | **0.006** | 0.082 | **0.038** | 0.272 |
| Subgroup_I_T1 vs. Controls | 495 | **0.042** | **0.044** | 0.079 | **0.004** | **0.002** | **0.010** | **0.012** | **0.014** |
| Subgroup_N_T1 vs. Controls | 495 | −0.776 | 0.482 | 0.397 | 0.258 | 0.069 | 0.536 | 0.242 | −0.502 |

$N_{all}$ is the number of all possible permutations.
The results of the statistical analysis are reported as *p*-values in the table.
For X vs. Y, a negative sign before a *p*-value indicates X < Y.
Bolded *p*-values indicate significant differences.
*p*-value with underline indicates the minimum obtainable *p*-value is achieved.

## Supplementary Table 8. Results of analysis of the difference in functional connectivity.

| | *N* | *δ* | | *θ* | | *α* | | *β* | | *γ* | |
|---|---|---|---|---|---|---|---|---|---|---|---|
| | | **DEC** | **INC** | **DEC** | **INC** | **DEC** | **INC** | **DEC** | **INC** | **DEC** | **INC** |
| Patients_T1 vs. Patients_T0 | 256 | 1.000 | 0.156 | 0.202 | 1.000 | 0.070 | 1.000 | 1.000 | 0.358 | 1.000 | 1.000 |
| Subgroup_I_T0 vs. Subgroup_N_T0 | 70 | 1.000 | 1.000 | 1.000 | 0.479 | 1.000 | 0.169 | 0.761 | 1.000 | 0.817 | 1.000 |
| Subgroup_I_T1 vs. Subgroup_N_T1 | 70 | 1.000 | 0.648 | 1.000 | 0.056 | 0.732 | 1.000 | 0.820 | 1.000 | 1.000 | 0.282 |
| Patients_T0 vs. Controls | 2000 | 0.881 | 0.200 | 1.000 | 0.091 | 0.078 | 0.898 | 0.130 | 0.679 | 1.000 | 0.077 |
| Patients_T1 vs. Controls | 2000 | 0.964 | 0.099 | 0.878 | 0.186 | **0.018** | 1.000 | 0.381 | 0.314 | 1.000 | 0.119 |

*N* is the number of permutations.
The results of the statistical analysis are reported as *p*-values in the table. Only the smallest *p*-value among the connected components is provided.
Bolded *p*-values indicate significant differences.
DEC: decrease; INC: increase.
For subgroups comparisons, the univariate comparisons were used permutation tests.

## Supplementary Table 9. Group average statistics (±*SD*) of the Patients at T0, Patients at T1, and Healthy controls for all the computed microstates parameters and classes.

| **Parameters** | **Microstate** | **Patients_T0** | **Patients_T1** | **Controls** |
|---|---|---|---|---|
| GEV (%) | Class A | 15.263 ± 3.668 | 14.459 ± 3.385 | 7.516 ± 2.967 |
| | Class B | 15.716 ± 5.032 | 18.225 ± 5.505 | 5.975 ± 2.239 |
| | Class C | 17.975 ± 3.144 | 19.238 ± 9.080 | 31.150 ± 9.178 |
| | Class D | 7.583 ± 3.565 | 7.036 ± 3.613 | 5.206 ± 1.963 |
| Mean Duration (ms) | Class A | 73.800 ± 9.082 | 84.338 ± 22.970 | 56.663 ± 4.907 |
| | Class B | 76.900 ± 14.834 | 92.050 ± 19.215 | 56.075 ± 4.385 |
| | Class C | 80.663 ±14.331 | 97.538 ± 40.746 | 101.025 ± 21.061 |
| | Class D | 68.875 ± 19.698 | 79.138 ± 26.138 | 55.488 ± 4.336 |
| Occurrence (Hz) | Class A | 3.456 ± 0.819 | 2.925 ± 0.774 | 3.095 ± 0.430 |
| | Class B | 3.459 ± 0.739 | 3.331 ± 1.149 | 2.881 ± 0.559 |
| | Class C | 3.765 ± 0.726 | 3.280 ± 0.938 | 4.856 ± 0.332 |
| | Class D | 2.771 ± 0.421 | 2.411 ± 0.528 | 3.076 ± 0.569 |
| Coverage (%) | Class A | 24.975 ± 4.172 | 23.213 ± 2.451 | 17.725 ± 4.014 |
| | Class B | 26.300 ± 6.089 | 28.950 ± 5.979 | 16.338 ± 4.268 |
| | Class C | 29.750 ± 4.165 | 29.463 ± 6.193 | 48.762 ± 8.778 |
| | Class D | 18.975 ±5.176 | 18.375 ± 5.494 | 17.200 ± 4.219 |
| Mean Interval (ms) | Class A | 233.500 ± 78.438 | 281.000 ± 80.644 | 272.125 ± 45.961 |
| | Class B | 194.000 ± 43.204 | 230.625 ± 64.334 | 303.750 ± 75.115 |
| | Class C | 300.375 ± 64.933 | 359.625 ± 117.090 | 106.475 ± 19.045 |
| | Class D | 228.875 ± 39.851 | 269.750 ± 78.964 | 279.375 ± 60.910 |
| Mean GFP (a.u.) | Class A | 0.944 ± 0.027 | 0.934 ± 0.030 | 0.890 ± 0.044 |
| | Class B | 0.937 ± 0.026 | 0.939 ± 0.045 | 0.861 ± 0.060 |
| | Class C | 0.951 ± 0.014 | 0.946 ± 0.049 | 0.994 ± 0.004 |
| | Class D | 0.859 ± 0.042 | 0.828 ± 0.073 | 0.846 ± 0.068 |

Mean ± standard deviation for each parameter is provided in the table.

## Supplementary Table 10. Results of analysis of the difference in microstate features.

| MS parameters | Microstate | Patients_T1 vs. Patients_T0 ($N_{all}$ = 256) | Subgroup_I_T1 vs. Subgroup_I_T0 ($N_{all}$ = 16) | Subgroup_N_T1 vs. Subgroup_N_T0 ($N_{all}$ = 16) | Subgroup_I_T0 vs. Subgroup_N_T0 ($N_{all}$ = 70) | Subgroup_I_T1 vs. Subgroup_N_T1 ($N_{all}$ = 70) |
|---|---|---|---|---|---|---|
| GEV | Class A | −0.572 | 0.882 | −0.412 | −0.690 | 0.831 |
| | Class B | 0.377 | 0.765 | 0.412 | 0.324 | 0.521 |
| | Class C | 0.907 | −0.059 | 0.294 | −0.380 | −0.099 |
| | Class D | −0.696 | 0.529 | −0.176 | −0.634 | 0.239 |
| Mean Duration | Class A | 0.167 | 0.176 | 0.882 | 0.887 | 0.408 |
| | Class B | **0.004** | 0.059 | 0.059 | 0.437 | 0.211 |
| | Class C | 0.113 | 0.176 | 0.765 | −0.803 | −0.915 |
| | Class D | 0.253 | 0.059 | −0.294 | −0.606 | 0.268 |
| Occurrence | Class A | **−0.012** | −0.059 | −0.176 | −0.775 | −0.408 |
| | Class B | −0.735 | −0.176 | 0.529 | 0.437 | −0.380 |
| | Class C | −0.058 | −0.059 | −0.529 | −0.634 | −0.183 |
| | Class D | −0.097 | −0.294 | −0.294 | −0.549 | −0.775 |
| Coverage | Class A | −0.339 | −0.765 | −0.412 | −0.718 | 0.465 |
| | Class B | 0.455 | −0.882 | 0.294 | 0.183 | 0.887 |
| | Class C | −0.860 | −0.176 | 0.647 | −0.380 | −0.127 |
| | Class D | −0.782 | 0.412 | −0.176 | −0.606 | 0.211 |
| Mean Interval | Class A | **0.004** | 0.059 | 0.059 | 0.887 | 0.690 |
| | Class B | 0.611 | 0.176 | −0.529 | −0.183 | 0.662 |
| | Class C | 0.066 | 0.059 | 0.647 | 0.521 | **0.042** |
| | Class D | 0.222 | 0.765 | 0.176 | 0.634 | −0.887 |
| Mean GFP | Class A | −0.300 | −0.647 | −0.059 | −0.972 | 0.775 |
| | Class B | 0.922 | 0.529 | −0.765 | 0.296 | 0.268 |
| | Class C | −0.798 | −0.176 | 0.176 | 0.352 | −0.127 |
| | Class D | −0.315 | −0.529 | −0.294 | −0.493 | 0.577 |

## Continued

| MS parameters | Microstate | Subgroup_I vs. Subgroup_N (Δ) ($N_{all}$ = 70) | Patients_T0 vs. Controls ($N_{all}$ = 12870) | Subgroup_I_T0 vs. Controls ($N_{all}$ = 495) | Subgroup_N_T0 vs. Controls ($N_{all}$ = 495) | Patients_T1 vs. Controls ($N_{all}$ = 12870) |
|---|---|---|---|---|---|---|
| GEV | Class A | 0.521 | **0.001** | **0.004** | **0.006** | **0.001** |
| | Class B | −0.803 | **0.001** | **0.002** | **0.010** | **0.000** |
| | Class C | **−0.042** | **−0.003** | **−0.016** | **−0.038** | **−0.024** |
| | Class D | 0.239 | 0.129 | 0.353 | 0.058 | 0.239 |
| Mean Duration | Class A | 0.324 | **2.33e−4** | **0.004** | **0.002** | **0.001** |
| | Class B | 0.408 | **0.001** | **0.002** | **0.014** | **7.77e−5** |
| | Class C | −0.887 | **−0.040** | −0.087 | −0.149 | −0.850 |
| | Class D | **0.014** | 0.061 | 0.113 | **0.014** | **0.032** |
| Occurrence | Class A | −0.408 | 0.281 | 0.427 | 0.272 | −0.591 |
| | Class B | −0.099 | 0.101 | 0.067 | 0.347 | 0.331 |
| | Class C | −0.155 | **−0.002** | **−0.004** | **−0.016** | **−0.001** |
| | Class D | 0.859 | −0.248 | −0.250 | −0.532 | **−0.026** |
| Coverage | Class A | 0.493 | **0.005** | **0.026** | **0.014** | **0.008** |
| | Class B | −0.493 | **0.003** | **0.004** | **0.044** | **0.001** |
| | Class C | −0.268 | **7.77e−5** | **−0.004** | **−0.004** | **−0.001** |
| | Class D | 0.099 | 0.462 | 0.808 | 0.290 | 0.642 |
| Mean Interval | Class A | 0.380 | −0.241 | −0.240 | −0.353 | 0.787 |
| | Class B | 0.324 | **−0.047** | **−0.044** | −0.246 | −0.238 |
| | Class C | **0.042** | **2.33e−4** | **0.002** | **0.002** | **7.77e−5** |
| | Class D | −0.465 | 0.533 | 0.401 | 0.935 | 0.078 |
| Mean GFP | Class A | 0.803 | **0.013** | 0.056 | 0.052 | **0.038** |
| | Class B | 0.634 | **0.008** | **0.028** | 0.081 | **0.013** |
| | Class C | **−0.042** | **7.77e−5** | **−0.002** | **-0.002** | **-0.013** |
| | Class D | 0.549 | 0.641 | 0.883 | 0.571 | −0.611 |

## Continued

| MS parameters | Microstate | Subgroup_I_T1 vs. Controls ($N_{all}$ = 495) | Subgroup_N_T1 vs. Controls ($N_{all}$ = 495) |
|---|---|---|---|
| GEV | Class A | **0.004** | **0.010** |
| | Class B | **0.002** | **0.002** |
| | Class C | **−0.010** | **−0.236** |
| | Class D | 0.063 | 0.899 |
| Mean Duration | Class A | **0.002** | **0.012** |
| | Class B | **0.002** | **0.002** |
| | Class C | −0.692 | −0.942 |
| | Class D | **0.010** | 0.139 |
| Occurrence | Class A | −0.226 | 0.867 |
| | Class B | 0.927 | 0.141 |
| | Class C | **−0.002** | **−0.008** |
| | Class D | **−0.030** | −0.171 |
| Coverage | Class A | **0.032** | 0.073 |
| | Class B | **0.004** | **0.004** |
| | Class C | **−0.002** | **−0.014** |
| | Class D | 0.224 | −0.500 |
| Mean Interval | Class A | 0.506 | −0.944 |
| | Class B | −0.442 | −0.234 |
| | Class C | **0.002** | **0.002** |
| | Class D | 0.111 | 0.137 |
| Mean GFP | Class A | 0.071 | 0.163 |
| | Class B | **0.020** | 0.125 |
| | Class C | **−0.002** | −0.288 |
| | Class D | −0.960 | −0.433 |

$N_{all}$ is the number of all possible permutations.
The results of the statistical analysis are reported as *p*-values in the table.
For X vs. Y, a negative sign before a *p*-value indicates X < Y.
Bolded *p*-values indicate significant differences.
*p*-value with underline indicates the minimum obtainable *p*-value is achieved.

## Supplementary Table 11. Classification performance of the linear kernel SVM models.

| Model | Accuracy | Sensitivity/Recall | Specificity |
|---|---|---|---|
| With PCA (the first two principal components) | 1.000 (16/16) | 1.000 (8/8) | 1.000 (8/8) |
| Without PCA | 0.875 (14/16) | 1.000 (8/8) | 0.750 (6/8) |

Samples after zolpidem administration are labeled as positive.

**Supplementary Table 12. Results of correlation analysis between spectral feature and CRS-R score at $T_{end}$.**

| $N_{all}$ = 40320 | | $\delta$ | $\theta$ | $\alpha$ | $\beta$ | Low $\beta$ | Middle $\beta$ | High $\beta$ | $\gamma$ |
|---|---|---|---|---|---|---|---|---|---|
| Relative power at T0–CRS-R | *p* | 0.669 | 0.605 | 0.804 | 0.711 | 0.502 | 0.502 | 0.781 | 0.941 |
| | *r* | 0.169 | −0.205 | 0.096 | −0.145 | −0.265 | −0.265 | −0.108 | −0.024 |
| Relative power at T1–CRS-R | *p* | 0.140 | 0.184 | 0.448 | **0.015** | 0.203 | **0.028** | **0.011** | 0.285 |
| | *r* | 0.566 | −0.518 | −0.301 | **−0.819** | −0.494 | **−0.771** | **−0.843** | −0.422 |
| pdBSI at T0–CRS-R | *p* | 0.426 | 0.230 | 0.140 | 0.464 | 0.502 | 0.342 | 0.669 | 0.758 |
| | *r* | 0.313 | 0.470 | 0.566 | 0.289 | 0.265 | 0.374 | 0.169 | 0.120 |
| pdBSI at T1–CRS-R | *p* | 0.711 | 0.758 | 0.242 | 0.314 | 0.393 | 0.242 | 0.541 | 0.052 |
| | *r* | 0.145 | 0.120 | 0.458 | 0.398 | 0.337 | 0.458 | 0.241 | 0.711 |
| rBSI at T0–CRS-R | *p* | **0.011** | **0.040** | 0.150 | 0.242 | 0.106 | 0.064 | 0.374 | 0.804 |
| | *r* | **0.843** | **0.735** | 0.554 | 0.458 | 0.615 | 0.687 | 0.349 | 0.096 |
| rBSI at T1–CRS-R | *p* | 0.314 | 0.203 | 0.170 | 0.068 | **0.028** | 0.106 | 0.242 | 0.150 |
| | *r* | 0.398 | 0.494 | 0.530 | 0.675 | **0.771** | 0.615 | 0.458 | 0.554 |

$N_{all}$ is the number of all possible permutations.
Bolded *p*-values and correlation coefficient indicate significant correlations.

**Supplementary Table 13. Results of correlation analysis between microstate feature and CRS-R score at $T_{end}$.**

| MS parameters | Microstate | $N_{all}$ | Feature_T0–CRS-R | | Feature_T1–CRS-R | |
|---|---|---|---|---|---|---|
| | | | *p* | *r* | *p* | *r* |
| GEV | Class A | 40320 | 0.693 | −0.157 | 0.150 | 0.558 |
| | Class B | 40320 | 0.605 | 0.205 | 0.485 | 0.277 |
| | Class C | 40320 | 0.132 | −0.578 | 0.170 | −0.530 |
| | Class D | 40320 | 0.485 | −0.277 | 0.242 | 0.458 |
| Mean Duration | Class A | 40320 | 0.693 | 0.157 | 0.150 | 0.554 |
| | Class B | 40320 | 0.605 | 0.205 | 0.140 | 0.566 |
| | Class C | 40320 | 0.669 | −0.169 | 0.541 | 0.241 |
| | Class D | 40320 | 0.892 | 0.048 | 0.230 | 0.470 |
| Occurrence | Class A | 40320 | 0.522 | −0.253 | 0.541 | −0.241 |
| | Class B | 40320 | 0.541 | 0.241 | 0.325 | −0.386 |
| | Class C | 40320 | 0.565 | −0.229 | 0.170 | −0.530 |
| | Class D | 40320 | 0.889 | −0.055 | 0.162 | −0.542 |
| Coverage | Class A | 40320 | 0.711 | −0.145 | **0.036** | **0.747** |
| | Class B | 40320 | 0.285 | 0.422 | 0.821 | −0.091 |
| | Class C | 40320 | 0.068 | −0.675 | 0.112 | −0.606 |
| | Class D | 40320 | 0.846 | −0.072 | 0.249 | 0.455 |
| Mean Interval | Class A | 40320 | 0.565 | 0.229 | 0.541 | 0.241 |
| | Class B | 40320 | 0.184 | −0.518 | 0.311 | 0.400 |
| | Class C | 40320 | 0.314 | 0.398 | **0.008** | **0.855** |
| | Class D | 40320 | 0.892 | −0.048 | 0.411 | 0.325 |
| Mean GFP | Class A | 40320 | 0.804 | 0.096 | 0.586 | 0.220 |
| | Class B | 40320 | 0.314 | 0.398 | 0.464 | 0.289 |
| | Class C | 40320 | 0.902 | 0.049 | 0.058 | −0.699 |
| | Class D | 40320 | 0.565 | −0.229 | 0.892 | −0.048 |

$N_{all}$ is the number of all possible permutations.
Bolded *p*-values and correlation coefficient indicate significant correlations.

## Supplementary Table 14. Results of correlation analysis between functional connectivity and CRS-R score at $T_{end}$.

| | $N$ | $\delta$ | | $\theta$ | | $\alpha$ | | $\beta$ | | $\gamma$ | |
|---|---|---|---|---|---|---|---|---|---|---|---|
| | | NEG | POS | NEG | POS | NEG | POS | NEG | POS | NEG | POS |
| FC_T0–CRS-R | 2000 | 0.931 | 1.000 | 1.000 | 1.000 | 1.000 | 0.174 | 1.000 | 1.000 | 1.000 | 1.000 |
| MFCSCC_T0–CRS-R | 40320 | | | | | | **2.48e-5** | | | | |
| FC_T1–CRS-R | 2000 | 1.000 | 0.359 | 1.000 | 0.311 | 1.000 | 1.000 | 0.547 | 1.000 | 1.000 | 0.452 |
| MFCSCC_T1–CRS-R | 40320 | | | | **0.002** | | | | | | |

*N* is the number of permutations.
The results of the correlation analysis are reported as *p*-values in the table. Bolded *p*-values indicate significant correlations.
NEG: negative correlation; POS: positive correlation.
FC: functional connectivity; MFCSCC: mean functional connectivity strength of the connected component with the smallest *p*-value.

## Supplementary Table 15. Prediction performance of the linear kernel SVR models.

| **Model** | **1** | **2** | **3** | |
|---|---|---|---|---|
| **Feature used** | **$\alpha$-MFCSCC at T0** | **$\theta$-MFCSCC at T1** | **PC1 of $\alpha$-MFCSCC at T0 and $\theta$-MFCSCC at T1** | |
| | **Predicted score** | **Predicted score** | **Predicted score** | **CRS-R score at $T_{end}$** |
| Score of each patient | 12.151 | 10.283 | 11.212 | 22 |
| | 11.378 | 14.052 | 12.507 | 12 |
| | 8.096 | 7.733 | 7.741 | 8 |
| | 12.705 | 10.942 | 11.841 | 12 |
| | 5.592 | 6.047 | 6.126 | 6 |
| | 7.300 | 6.152 | 6.757 | 6 |
| | 9.068 | 9.543 | 9.128 | 10 |
| | 7.399 | 7.448 | 7.357 | 7 |
| #Permutation | 40320 | 40320 | 40320 | |
| *p* | 0.001 | 0.002 | 0.002 | |
| *r* | 0.952 | 0.916 | 0.916 | |
| RMSE with outlier | 3.549 | 4.230 | 3.844 | |
| RMSE without outlier | 0.735 | 0.913 | 0.511 | |

RMESE: root-mean-square error; PC1: the first principal component; MFCSCC: mean functional connectivity strength of the connected component with the smallest *p*-value.

## 3 Supplementary Figures

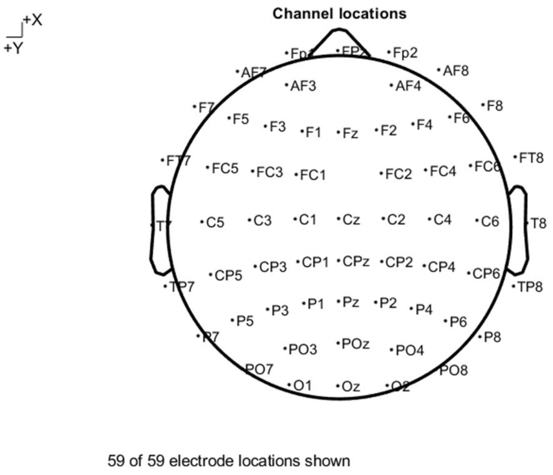


**Supplementary Figure 1. The electrode placements of the EEG data of patients.**

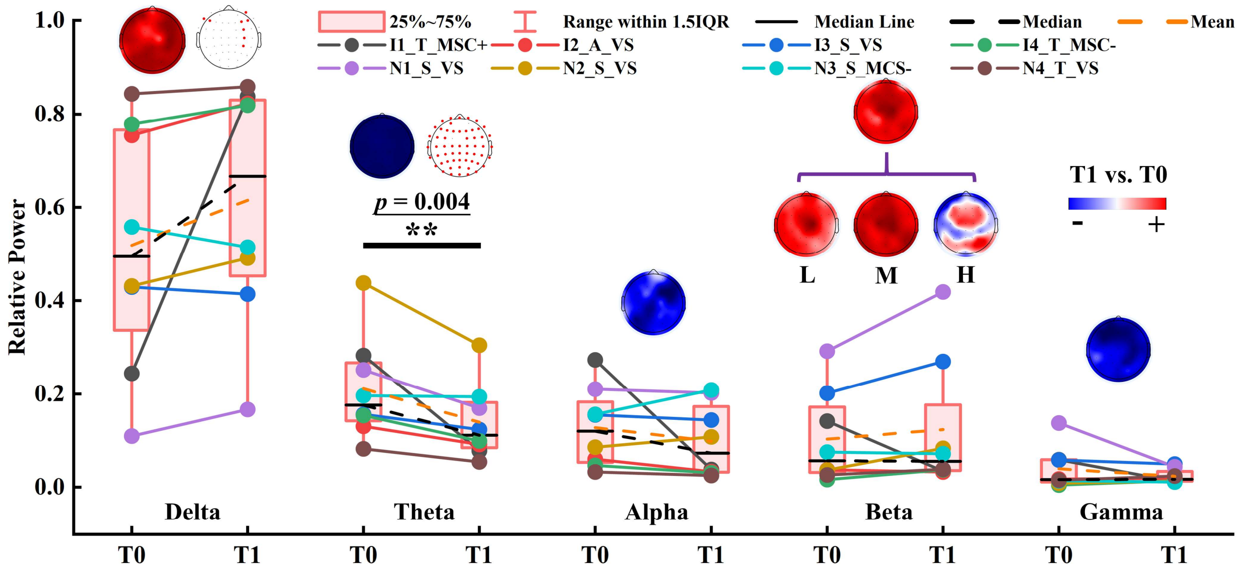


**Supplementary Figure 2. Differences between T0 and T1 in relative power.** The box line plot demonstrates the change in the average relative power of the whole brain. The topographic map above the box plot shows the change in relative power at the electrode level before and after zolpidem administration, and if there is a significant difference, it is marked with a red dot in the other topographic map. Red and blue colors indicate higher, lower relative power for patients at T1 versus patients at T0, respectively. L, M, and H denote the low $\beta$-band, middle $\beta$-band, and high $\beta$-band, respectively. $^{**}p < 0.01$; $p$-value with underline indicates the minimum obtainable $p$-value is achieved.

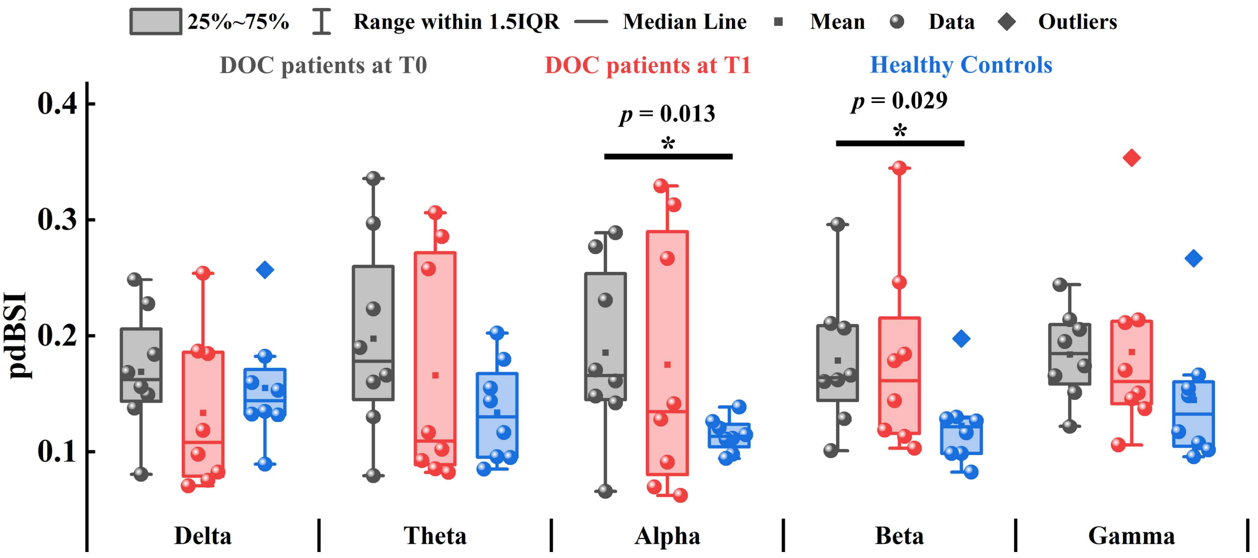


**Supplementary Figure 3. pdBSI of DOC patients and healthy controls.** *$p < 0.05$.

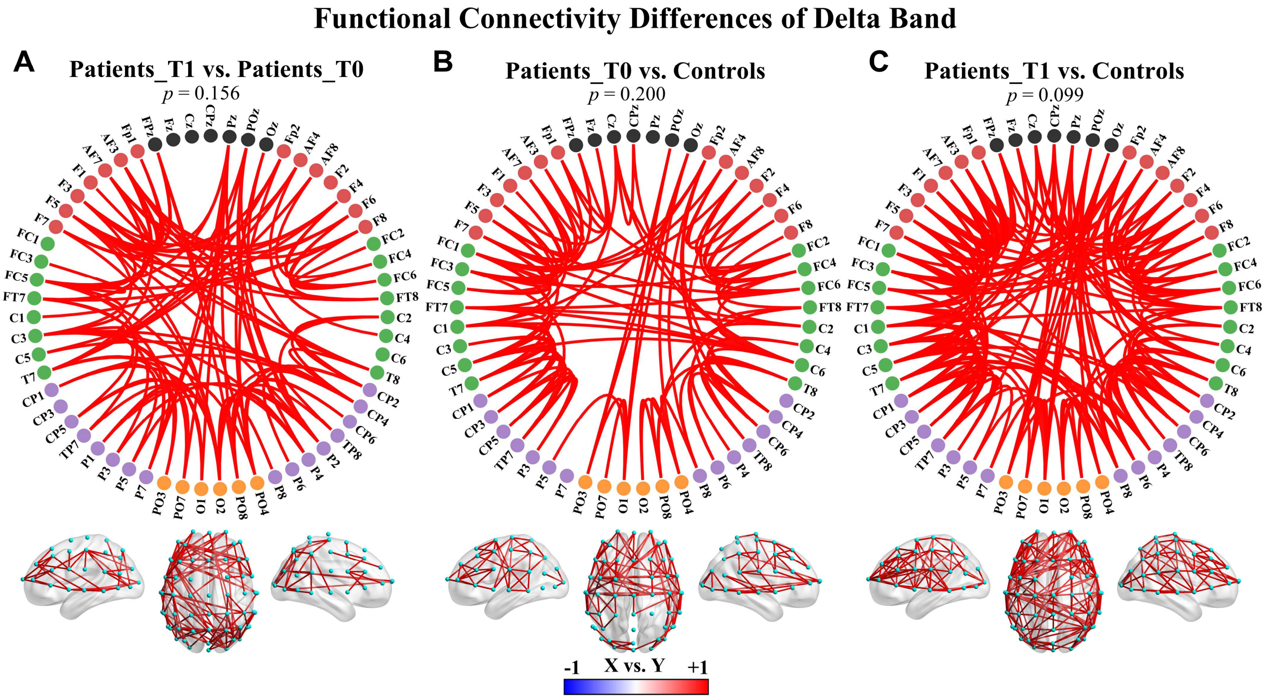


**Supplementary Figure 4. Group differences in functional connectivity of the delta band.** (**A**) Patients at T1 vs. Patients at T0. (**B**) Patients at T0 vs. Controls. (**C**) Patients at T1 vs. Controls. The depth of color indicates the size of the connectivity difference. Only the connected component with the smallest *p*-value is displayed.

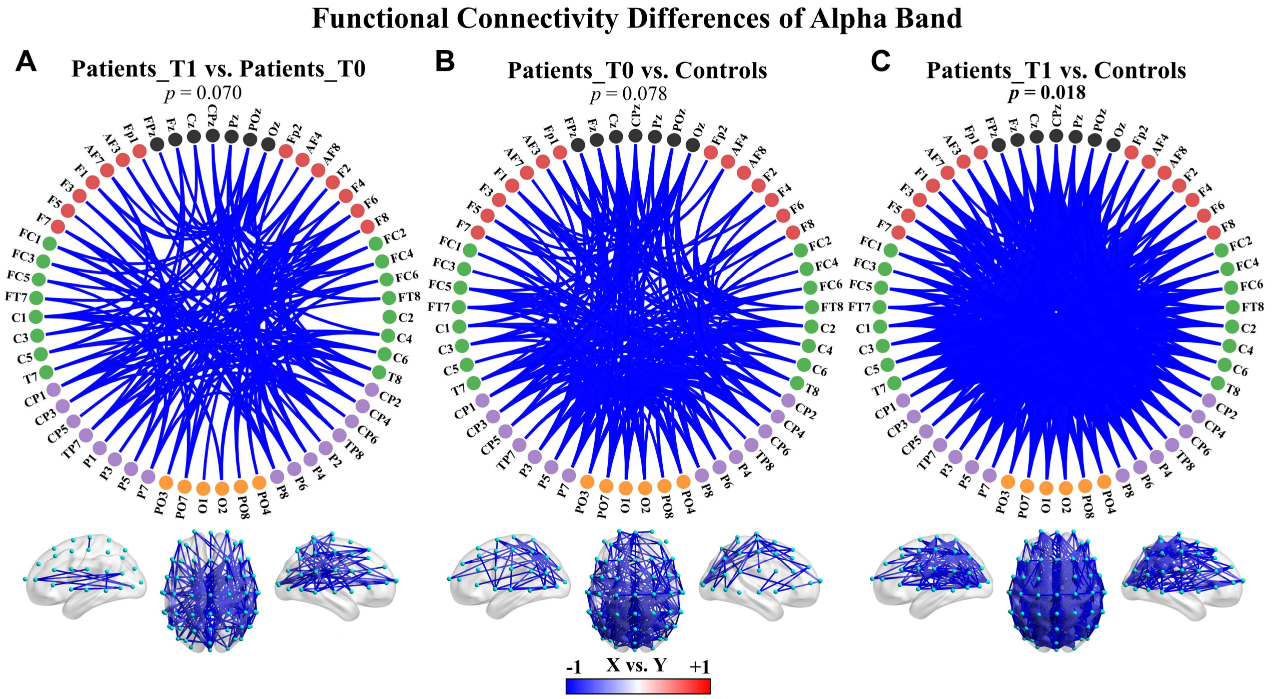


**Supplementary Figure 5. Group differences in functional connectivity of the alpha band.** (**A**) Patients at T1 vs. Patients at T0. (**B**) Patients at T0 vs. Controls. (**C**) Patients at T1 vs. Controls. The depth of color indicates the size of the connectivity difference. For X vs. Y, the blue color indicates X < Y; the red color indicates X > Y. Only the connected component with the smallest *p*-value is displayed. Bolded *p*-values indicate significant differences.

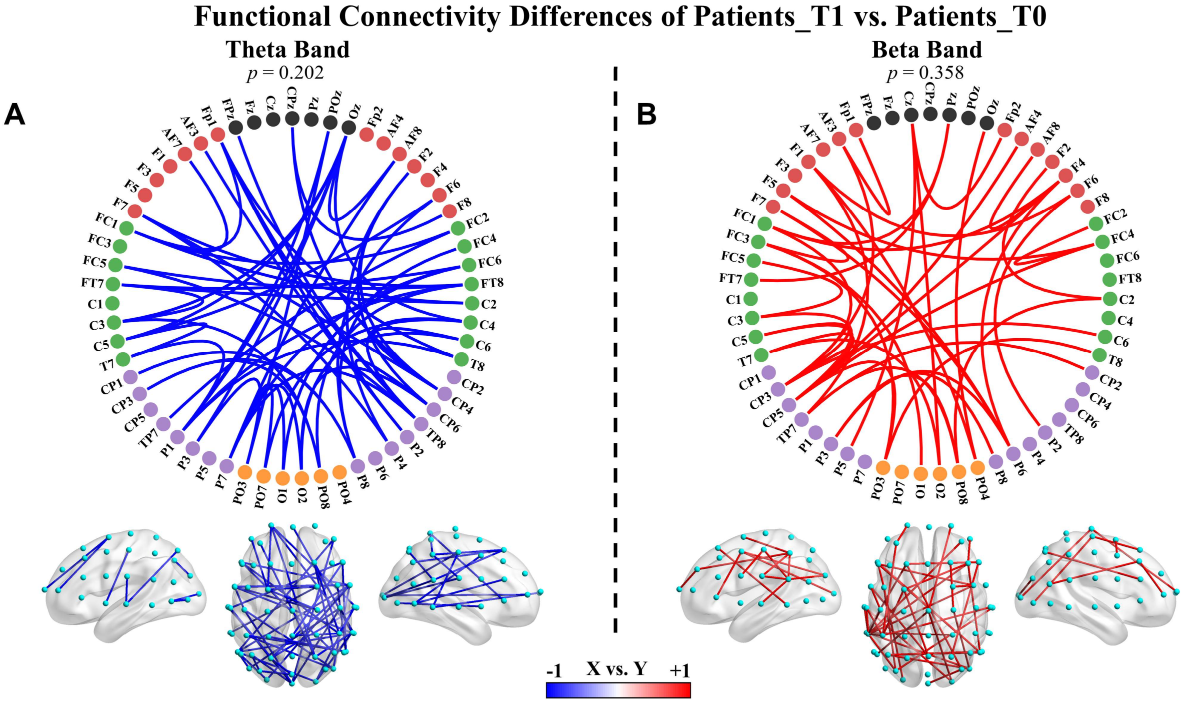


**Supplementary Figure 6. Group differences in functional connectivity between patients at T0 and patients at T1.** (**A**) Theta band. (**B**) Beta band. The depth of color indicates the size of the connectivity difference. For X vs. Y, the blue color indicates X < Y; the red color indicates X > Y. Only the connected component with the smallest *p*-value is displayed.

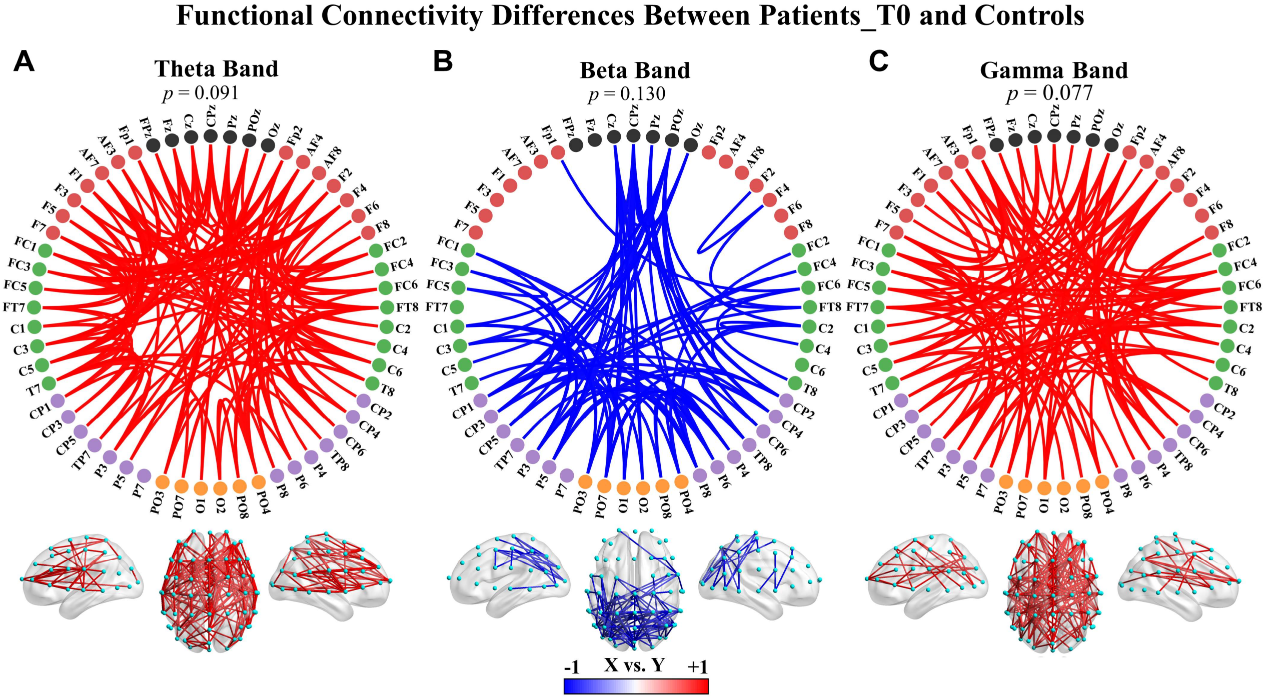


**Supplementary Figure 7. Group differences between patients at T0 and healthy controls in functional connectivity.** (**A**) Theta band. (**B**) Beta band. (**C**) Gamma band. The depth of color indicates the size of the connectivity difference. For X vs. Y, the blue color indicates X < Y; the red color indicates X > Y. Only the connected component with the smallest *p*-value is displayed.

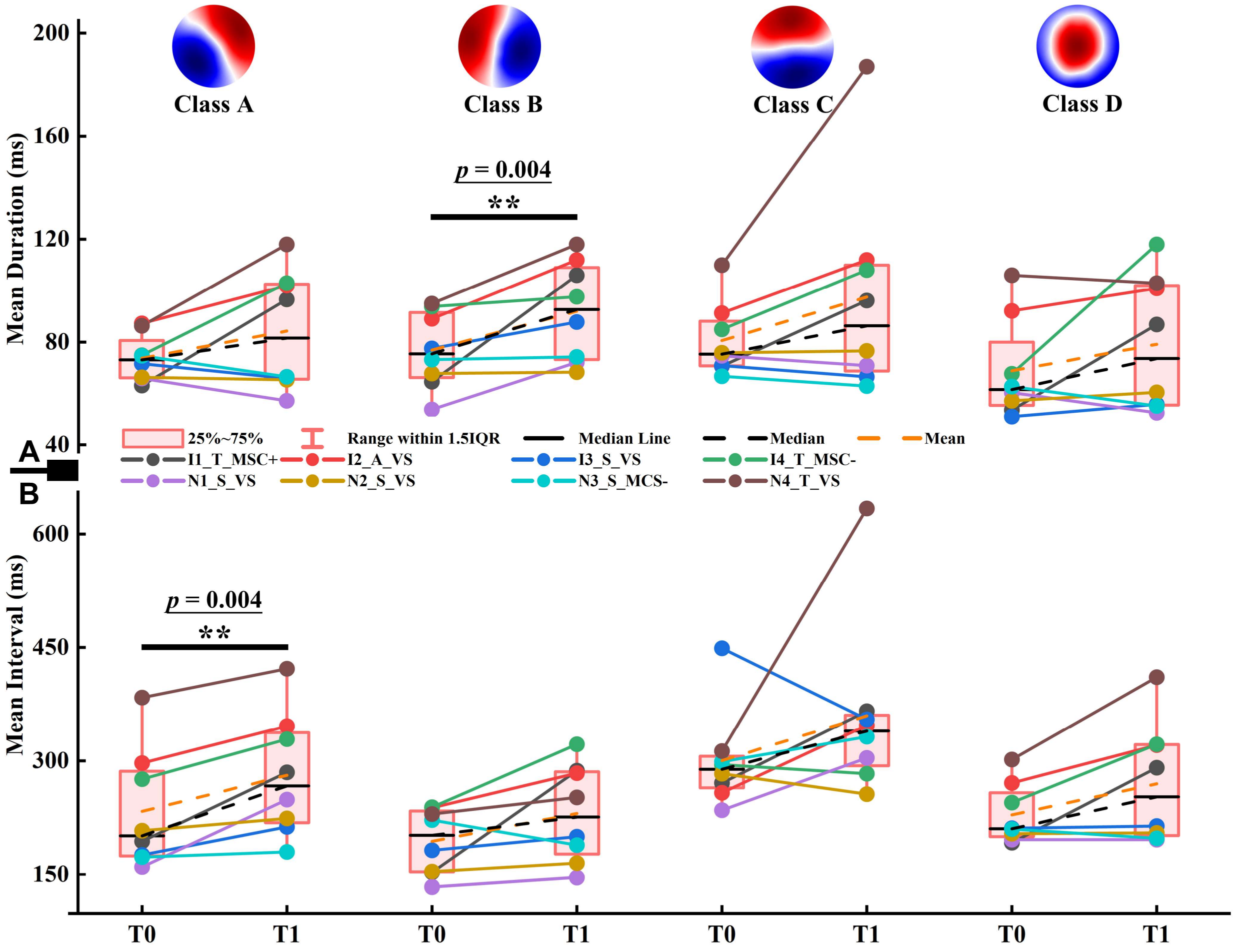


**Supplementary Figure 8. Microstate templates and differences between patients at T0 and T1 in microstate features.** (**A**) Mean duration. (**B**) Mean interval. The box line plot depicts the change in microstate features from T0 to T1. Four microstate templates of DOC patients are shown at the top of the figure. $**p < 0.01$; *p*-value with underline indicates the minimum obtainable *p*-value is achieved.

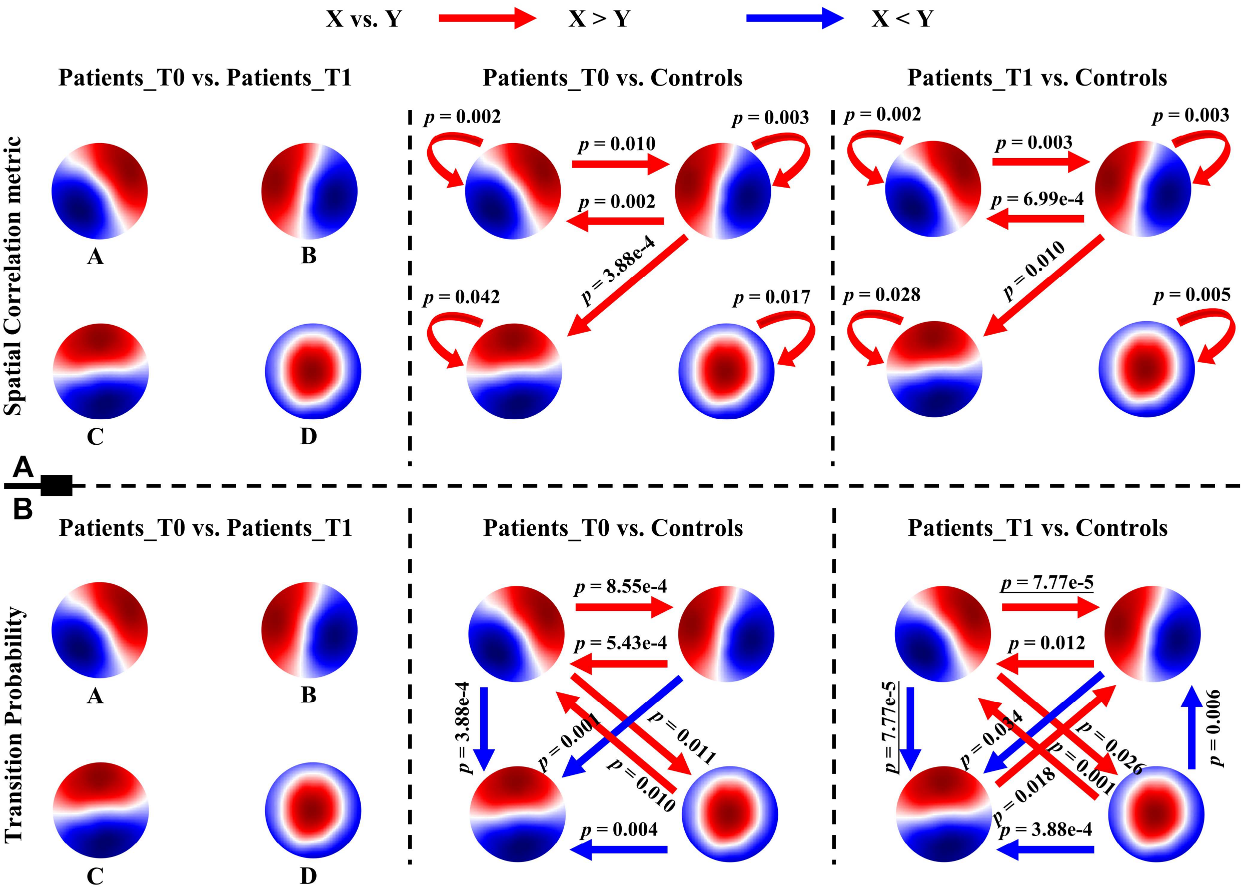


**Supplementary Figure 9. Results of comparisons in spatial correlation and transition probability features.** (**A**) Spatial correlation metric. (**B**) Transition probability. *p*-value with underline indicates the minimum obtainable *p*-value is achieved.

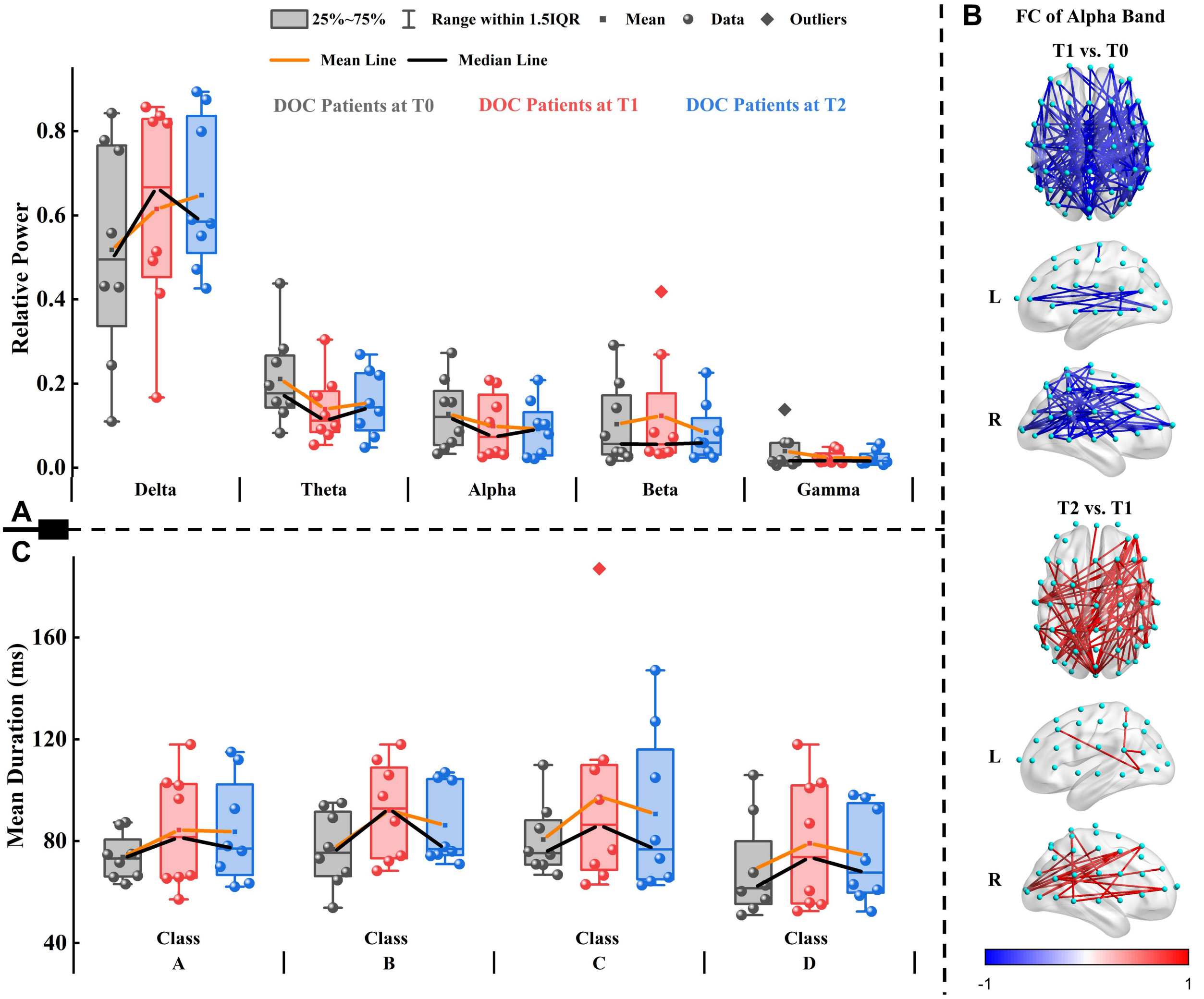


**Supplementary Figure 10. Dynamic changes of EEG features from T0 to T2.** (**A**) pdBSI. (**B**) Mean duration. (**C**) Functional connectivity of alpha band. FC: functional connectivity. For X vs. Y, the blue color indicates X < Y; the red color indicates X > Y.

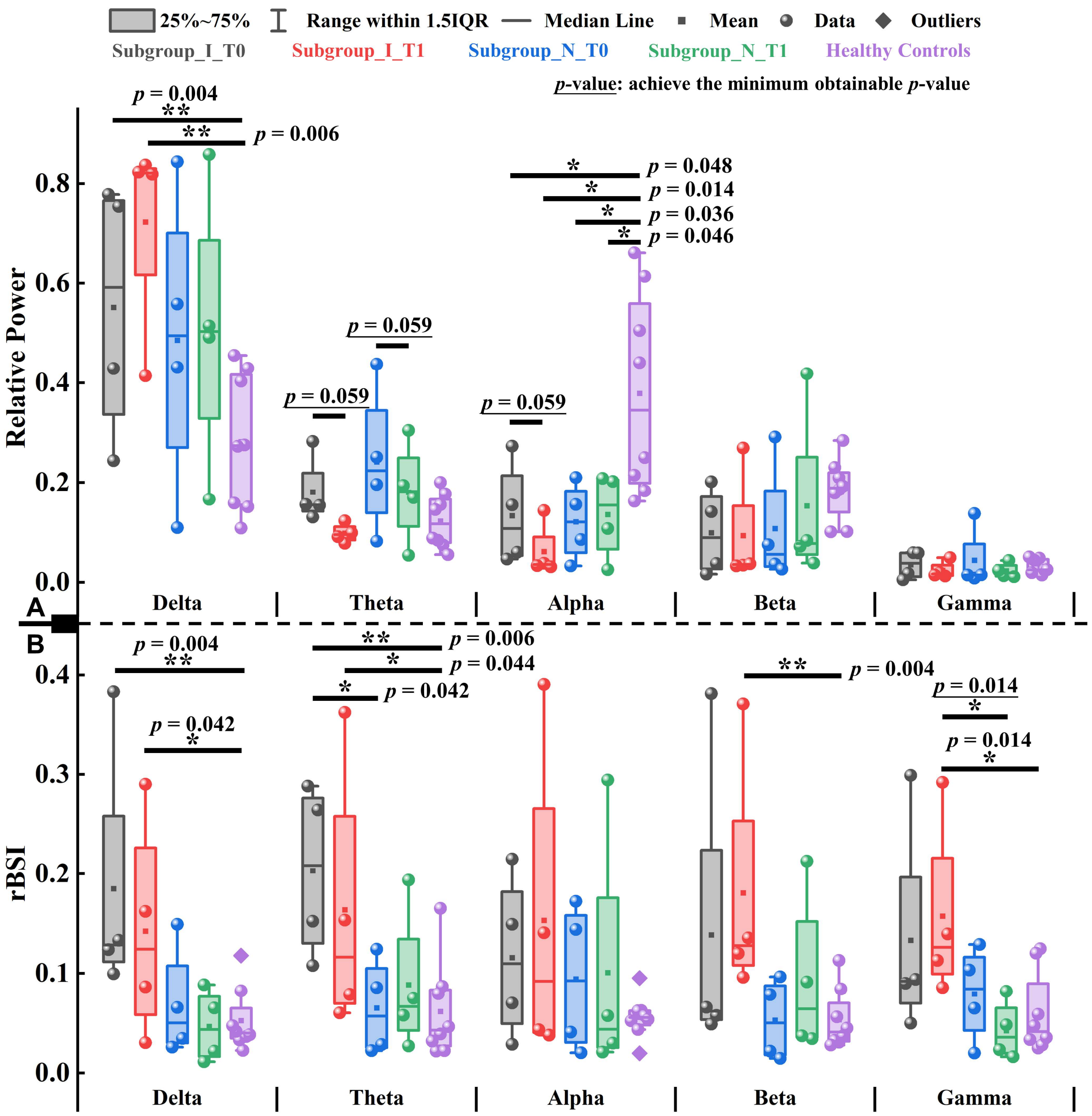


**Supplementary Figure 11. Comparisons between subgroups with improvement (Subgroup_I) and with non-improvement (Subgroup_N) in spectral features.** (**A**) Relative power. (**B**) rBSI. *$p < 0.05$; **$p < 0.01$; $p$-value with underline indicates the minimum obtainable $p$-value is achieved.

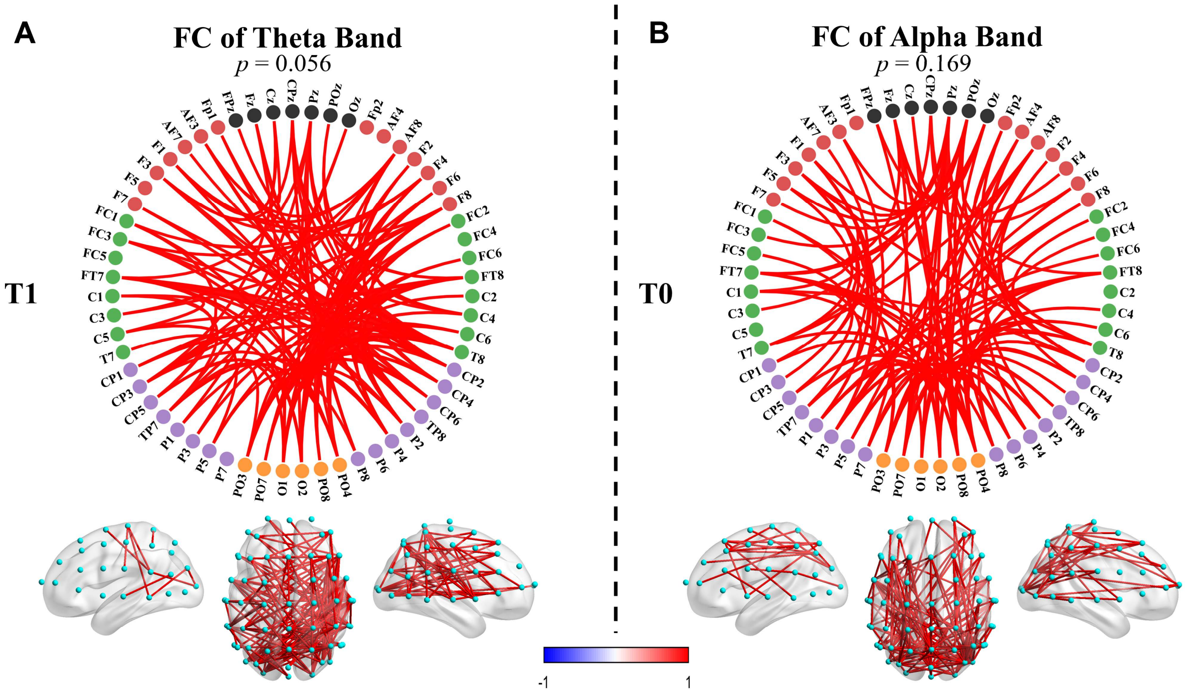


**Supplementary Figure 12. Group differences in functional connectivity for Subgroup_I vs. Subgroup_N.** (**A**) Subgroup_I vs. Subgroup_N at T1 of the theta band. (**B**) Subgroup_I vs. Subgroup_N at T1 of the alpha band. The depth of color indicates the size of the connectivity difference. For X vs. Y, the blue color indicates X < Y; the red color indicates X > Y. Only the connected component with the smallest *p*-value is displayed.

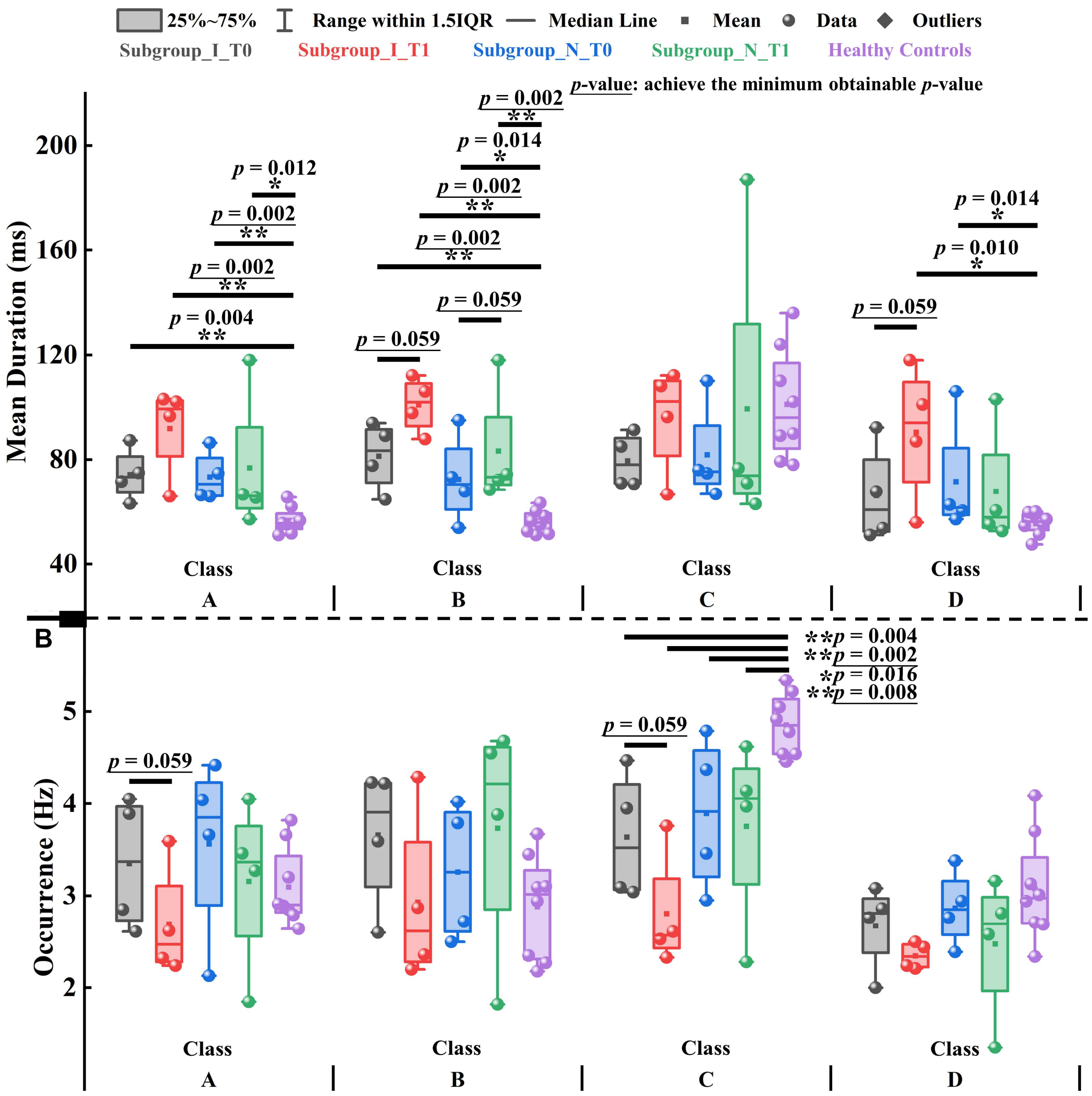


**Supplementary Figure 13. Comparisons between Subgroup_I and Subgroup_N in microstate features.** (**A**) Mean duration. (**B**) Occurrence. *$p$ < 0.05; **$p$ < 0.01; $p$-value with underline indicates the minimum obtainable $p$-value is achieved.

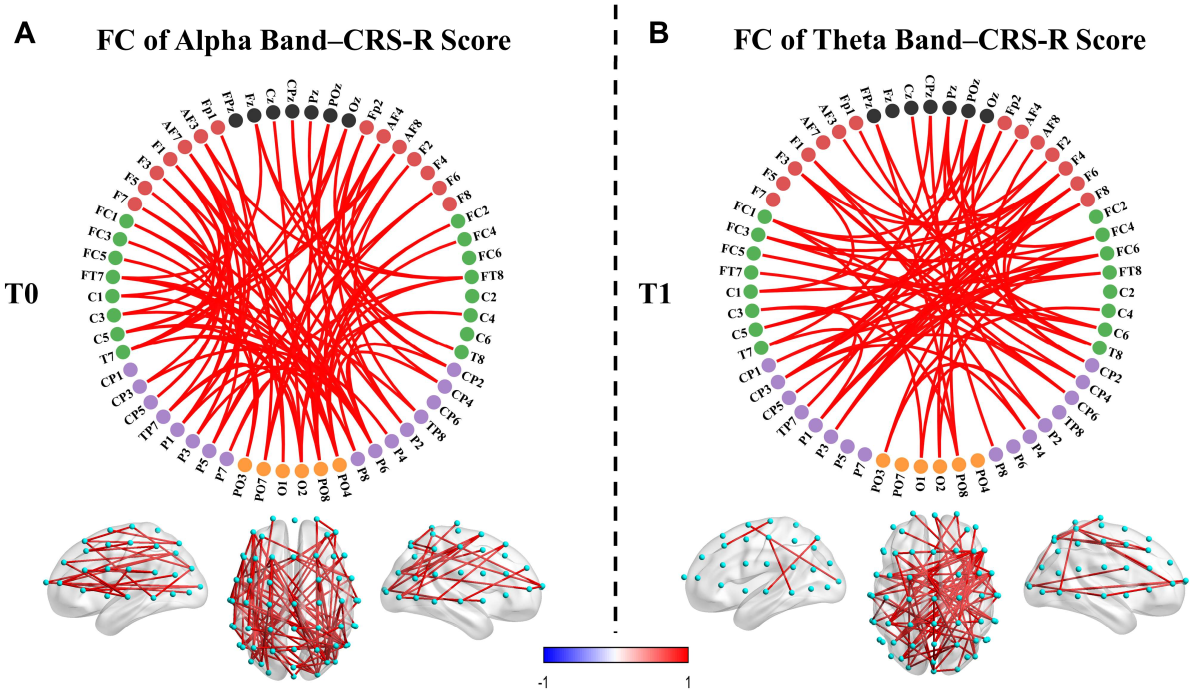


**Supplementary Figure 14. Correlation between functional connectivity and CRS-R score at $T_{end}$.** (**A**) Correlation between alpha-band functional connectivity at T0 and CRS-R score at $T_{end}$. (**B**) Correlation between theta-band functional connectivity at T1 and CRS-R score at $T_{end}$. The depth of the color indicates the degree of correlation. The red color indicates positive correlations. Only the connected component with the smallest *p*-value is displayed. FC: functional connectivity.
